# High Prevalence of Beta-Lactam-Resistant *Escherichia coli* in South Australian Grey-Headed Flying Fox Pups (*Pteropus poliocephalus*)

**DOI:** 10.3390/microorganisms10081589

**Published:** 2022-08-07

**Authors:** Fiona McDougall, Wayne Boardman, Michelle Power

**Affiliations:** 1 School of Natural Sciences, Macquarie University, Sydney, NSW 2109, Australia; 2 School of Animal and Veterinary Sciences, University of Adelaide, Adelaide, SA 5371, Australia

**Keywords:** antimicrobial resistance, bats, zoonoses, one health, bacterial pathogens, wildlife rehabilitation, antimicrobial stewardship

## Abstract

The emergence of antimicrobial-resistant *Escherichia coli* in wildlife is concerning—especially resistance to clinically important beta-lactam antibiotics. Wildlife in closer proximity to humans, including in captivity and in rescue/rehabilitation centres, typically have a higher prevalence of antimicrobial-resistant *E. coli* compared to their free-living counterparts. Each year, several thousand Australian fruit bat pups, including the grey-headed flying fox (GHFF; *Pteropus poliocephalus)*, require rescuing and are taken into care by wildlife rescue and rehabilitation groups. To determine the prevalence of beta-lactam-resistant *E. coli* in rescued GHFF pups from South Australia, faecal samples were collected from 53 pups in care. A combination of selective culture, PCR, antimicrobial susceptibility testing, whole-genome sequencing, and phylogenetic analysis was used to identify and genetically characterise beta-lactam-resistant *E. coli* isolates. The prevalence of amoxicillin-, amoxicillin-plus-clavulanic-acid-, and cephalosporin-resistant *E. coli* in the 53 pups was 77.4% (*n* = 41), 24.5% (*n* = 13), and 11.3% (*n* = 6), respectively. GHFF beta-lactam-resistant *E. coli* also carried resistance genes to aminoglycosides, trimethoprim plus sulphonamide, and tetracyclines in 37.7% (*n* = 20), 35.8% (*n* = 19), and 26.4% (*n* = 14) of the 53 GHFF pups, respectively, and 50.9% (*n* = 27) of pups carried multidrug-resistant *E. coli*. Twelve *E. coli* strain types were identified from the 53 pups, with six strains having extraintestinal pathogenic traits, indicating that they have the potential to cause blood, lung, or wound infections in GHFFs. Two lineages—*E. coli* ST963 and ST58 O8:H25—were associated with human extraintestinal infections. Phylogenetic analyses determined that all 12 strains were lineages associated with humans and/or domestic animals. This study demonstrates high transmission of anthropogenic-associated beta-lactam-resistant *E. coli* to GHFF pups entering care. Importantly, we identified potential health risks to GHFF pups and zoonotic risks for their carers, highlighting the need for improved antibiotic stewardship and biosafety measures for GHFF pups entering care.

## 1. Introduction

The emergence and global dissemination of antimicrobial-resistant *Escherichia coli* is accelerating in human, domestic animal, and wildlife populations [1,2]. Of particular concern are multidrug-resistant *E. coli* exhibiting resistance to clinically important antibiotics, including broad-spectrum and extended-spectrum beta-lactams, carbapenems, and fluoroquinolones [3,4]. The rapid spread of antimicrobial resistance (AMR) in *E. coli* is largely attributed to mobile genetic elements (MGEs), such as plasmids and transposons, which facilitate the horizontal transfer of antimicrobial resistance genes between *E. coli* and other Gram-negative bacteria [5]. Class 1 integrons are capable of carrying diverse antimicrobial resistance genes and, via their association with MGEs, also play an important role in the emergence and dissemination of AMR in *E. coli* [6]. 

Although *E. coli* is generally a commensal enteric bacterium of humans and animals, many strains are opportunistic pathogens capable of causing extraintestinal infections—most frequently in the urinary tract, blood, and lungs [7,8,9]. Extraintestinal pathogenic *E. coli* (ExPEC) are assigned to specific pathotypes according to the infection site and/or host species [10], including uropathogenic *E. coli* (UPEC) in urinary tract infections (UTIs), sepsis-associated *E. coli* (SEPEC), neonatal meningitis *E. coli* (NMEC), and avian pathogenic *E. coli* (APEC) [10]. ExPEC pathogenicity is associated with the carriage of specific combinations of virulence factors (VFs) that allow bacteria to colonise extraintestinal sites and cause disease [10]. Other *E. coli* strains are intestinal pathogens and are a significant cause of diarrhoeal disease in both humans [11] and animals [12]. Intestinal pathogenic *E. coli* (IPEC) are assigned to specific pathotypes according to mechanisms of disease and the presence of specific VFs, including enterohaemorrhagic *E. coli* (EHEC), enteropathogenic *E. coli* (EPEC), enterotoxigenic *E. coli* (ETEC) and Shiga-toxin-producing *E. coli* (STEC) [9].

Over the past decade, reports of antimicrobial-resistant *E. coli*, including isolates exhibiting resistance to clinically important antibiotics, have increased in wildlife species worldwide [13,14,15,16]. In Australian wildlife, antimicrobial-resistant *E. coli* have been detected in marine mammals [17,18], diverse terrestrial mammals [19,20], and birds [21,22,23,24,25]. Concerningly, *E. coli* exhibiting resistance to clinically important beta-lactam, carbapenem, and fluoroquinolone antibiotics have been detected in wild Australian birds [23,24,25] and mammals [19,20]. 

Whole-genome sequencing (WGS) and phylogenetic analysis of antimicrobial-resistant *E. coli* from wild animals indicates that isolates have frequently originated from anthropogenic sources, with many belonging to lineages associated with extraintestinal disease in humans, livestock, and companion animals [15,19,23,26]. The transmission of anthropogenic antimicrobial-resistant *E. coli* to wildlife is also demonstrated by the higher prevalence in wildlife species living and/or feeding in close proximity to urban environments [13,14,27,28]. Wildlife most likely acquire antimicrobial-resistant *E. coli* via exposure to environmental contamination, including from human and domestic animal effluent, wastewater treatment plants, agriculture runoff, and aquaculture farms [29]. Additionally, wildlife species living in captivity or rehabilitation facilities frequently harbour increased levels of antimicrobial-resistant *E. coli* compared to their free-living counterparts [22,30], due to increased exposure to sources of anthropogenic antimicrobial-resistant bacteria in captive environments compared to their wild environment [27].

Bats belong to the order Chiroptera, with fruit bats and flying foxes included in the suborder Yinpterochiroptera, and microbats predominantly included in the suborder Yangochiroptera [31]. *E. coli* exhibiting resistance to extended-spectrum beta-lactam antibiotics have been reported in African fruit bats [32,33] and in microbats from Peru [34], Portugal [35], and Nigeria [36]. The grey-headed flying fox (GHFF; *Pteropus poliocephalus*) is a large fruit bat species endemic to eastern Australia, congregating in tree roosts, or colonies, comprising up to 50,000 individual bats [37]. GHFF colonies are frequently located in urban environments, including botanic parks in major cities [38,39,40]. The IUCN Red List of Threatened Species currently lists GHFFs as “vulnerable to extinction” (https://www.iucnredlist.org/ (accessed on 24 July 2022)). *E. coli* exhibiting resistance to beta-lactam antibiotics were previously isolated from 3.5% of wild and 6.5% of captive adult GHFFs, with one *E. coli* isolate also exhibiting resistance to carbapenem and fluoroquinolone antibiotics [19]. WGS and phylogenetic analysis indicated that all antimicrobial-resistant *E. coli* isolates from adult GHFFs were anthropogenic-associated lineages, and analysis of virulence factors determined that 69.2% exhibited ExPEC characteristics [19].

In 2011, GHFFs established a new colony in the Adelaide Botanic Park (ABP), located in the city of Adelaide, South Australia [40]. The colony has since become permanently occupied, with the population increasing steadily to approximately 30,000 bats, over the past six years [41]. GHFF pups are typically born between October and December each year, and are carried by their mothers for their first month of life [42]. During GHFF pup season (approximately October to February each year), up to several hundred orphaned, abandoned, or heat-stress-affected pups are rescued by South Australian wildlife rescue and rehabilitation groups, and are taken into care until they reach juvenile age and are deemed suitable for release back into the wild. This study investigated the prevalence of beta-lactam-resistant *E. coli* in GHFF pups from the ABP colony that were taken into care during the 2018–2019 pup season. Beta-lactam-resistant *E. coli* underwent genetic characterisation to identify antimicrobial resistance genes and VFs associated with the ExPEC and IPEC pathotypes, along with phylogenetic analysis to identify anthropogenic-associated lineages.

## 2. Materials and Methods

### 2.1. Collection of Faecal Samples and Associated Metadata from GHFF Pups

Faecal samples (*n* = 58) were collected from 53 orphaned, abandoned, or heat-stress-affected GHFF pups entering care, with 5 pups sampled twice—i.e., at entry to care and at exit from care (*n* = 10 faecal samples)—and 48 pups sampled once (*n* = 48 faecal samples) (Supplementary Data S1). Pups entered care between 7 November 2018 and 24 January 2019, and samples were collected between 15 November 2018 and 27 January 2019 by a veterinarian or wildlife rescue and rehabilitation personnel from Fauna Rescue of South Australia (Supplementary Data S1). Sample collections were conducted with the approval of the animal ethics committees at Macquarie University (No. 2017/013) and the University of Adelaide (No. S-2015-028), along with a South Australia Department of Environment and Water Wildlife Scientific Permit (M-23671-1,2 and 3). Faecal samples were collected using the FecalSwab^TM^ system (COPAN, Brescia, Italy) from clean materials placed under flying fox pups (*n* = 18) or by rectal swab during veterinary examination (*n* = 40). FecalSwab samples were stored at 4 °C and then delivered to the laboratory for processing at room temperature. Received FecalSwab samples that were not immediately processed were stored at −80 °C with 35% glycerol until cultured. Metadata were collected for all 53 pups, including date of entry to care, animal weight and forearm length (FAL) measurement, medical history, reason for entry to care, and sampling data (Supplementary Data S1). GHFF pup age was estimated by correlating FAL with the Pinson and Kerr ageing chart [43].

### 2.2. Detection of Amoxicillin-Resistant E. coli in Faecal Samples

Faecal samples (*n* = 58) were cultured in media supplemented with amoxicillin to detect beta-lactam-resistant *E. coli*. FecalSwab medium (0.2 mL) was inoculated into 5 mL of Luria–Bertani (LB) broth (Difco Laboratories, Detroit, MI, USA) supplemented with 10 mg/L amoxicillin (Sigma, St. Louis, MO, USA) and incubated at 37 °C overnight. The LB broth was subsequently streaked onto Chromocult Coliform Agar (Merck Millipore, Burlington, MA, USA) supplemented with 10 mg/L amoxicillin (Sigma, St. Louis, MO, USA) and incubated at 37 °C overnight. Dark blue or purple colonies were presumptively identified as *E. coli*, and the corresponding faecal samples were deemed positive for the presence of amoxicillin-resistant *E. coli*. A single *E. coli* colony was selected from each positive agar plate, except where morphologically distinct colonies were observed (as determined by colony and halo colour), in which case one representative isolate was selected for each colony type observed.

### 2.3. Phylotyping and Class 1 Integron Screening of Amoxicillin-Resistant E. coli Isolates

GHFF isolates (*n* = 49) were inoculated into 10 mL of LB broth (Difco Laboratories, Detroit, MI, USA) and incubated at 37 °C overnight. Genomic DNA was extracted using the ISOLATE II Genomic DNA Kit (Bioline, London, UK), and DNA concentrations were determined using a Qubit dsDNA BR assay kit (Invitrogen, Waltham, MA, USA). 

The *E. coli* isolates were assigned to phylogroups (A, B1, B2, C, D, E, F, or cryptic clade I) using the Clermont quadruplex phylotyping PCR method [44]. Phylogroups were assigned based on the combination of four genes (*chuA, yjaA, tspE4.C2*, and *arpA*) [44]. PCR amplification was performed using GoTaq^®^ Green Master Mix (Promega, Madison, WI, USA), with all primers at 1 µM each, and cycling conditions of 94 °C for 4 min; 35 cycles at 94 °C for 20 s, 59 °C for 20 s, and 72 °C for 20 s; and a final extension at 72 °C for 4 min. 

Isolate DNA was screened for the class 1 integron integrase gene (*intI1*) using PCR with the primers HS463a and HS464, as previously described [19]. All *intI1-*positive isolates were then amplified using the primers HS458 and HS459 (to target the *attl1*-conserved and 3′ *qacE∆1*-conserved regions of the class 1 integron) [45] and the primers HS458 and JL-D2 (to target *attl1* and IS*26* transposase—an alternate 3′ sequence to the *qacE∆1*-conserved region) [46], using cycling conditions of 94 °C for 3 min; 35 cycles at 94 °C for 30 s, 55 °C for 30 s, and 72 °C for 1 min 30 sec; and a final extension at 72 °C for 5 min. The HS458/HS459 and HS458/JL-D2 PCRs both amplified the entire class 1 integron gene cassette array. 

### 2.4. ERIC and CGG PCRs of Amoxicillin-Resistant E. coli Isolates

To determine the strain types of all 49 *E. coli* isolates, genome fingerprints were obtained using enterobacterial repetitive intergenic consensus (ERIC) PCR [47] and CGG-PCR [48]. ERIC PCR amplification was performed using GoTaq^®^ Green Master Mix (Promega, Madison, WI, USA), ERIC1 and ERIC2 primers [47] at 0.5 µM each, 10 ng of DNA template, and cycling conditions of 95 °C for 7 min; 30 cycles of 94 °C for 1 min, 53 °C for 1 min, and 56 °C for 4 min; and a final extension at 65 °C for 16 min. CGG-PCR amplification was performed using GoTaq^®^ Green Master Mix (Promega, Madison, WI, USA), 0.5 µM N_6_(CGG)_4_ primer [48], 3.5 mM MgCl_2_, 10 ng of DNA template, and cycling conditions of 95 °C for 3 min; 35 cycles of 95 °C for 1 min and 72 °C for 3 min; and a final extension at 72 °C for 8 min. The PCR products underwent electrophoresis, and DNA fingerprints were visualised on 2% agarose gels (1 × TBE buffer) with SYBR Safe DNA Stain (Invitrogen, Waltham, MA, USA).

### 2.5. Whole-Genome Sequencing of E. coli Strains

All 49 *E. coli* isolates were assigned to one of 12 strain types using a combination of phylotyping, ERIC, CGG, and class 1 integron PCR data (Supplementary Table S1). For each *E. coli* strain type identified (*n* = 12), one representative isolate was selected to undergo whole-genome sequencing (WGS) (Supplementary Table S1). WGS was performed on an Illumina MiSeq system using the MiSeq reagent kit v2 (2 × 150 bp paired-end reads) at the Ramaciotti Centre for Genomics (Sydney, NSW, Australia). Raw sequence reads were assembled as de novo genome sequences using SPAdes Assembler 3.15.2 [49] in Geneious Prime 2022.0.2 (Biomatters Limited, Auckland, New Zealand). Raw sequence reads for all 12 *E. coli* isolates were uploaded to EnteroBase (http://enterobase.warwick.ac.uk/species/index/ecoli (accessed on 13 February 2021)) and the NCBI Sequence Read Archive (SRA) under BioProject PRJNA814458 (https://www.ncbi.nlm.nih.gov/bioproject/PRJNA814458 (accessed on 10 March 2022)). Individual isolate EnteroBase Barcodes and SRA accession numbers are provided in Supplementary Table S2.

Isolates uploaded to EnteroBase were assigned a sequence type (ST) using the Achtman seven-gene MLST scheme, predicted serotype (O:H), *fimH* type using fimTyper [50], and phylogroup using ClermonTyping [51] (available at http://enterobase.warwick.ac.uk/species/index/ecoli (accessed on 13 February 2021)) [52]. ST58 isolates were assigned to a sub-lineage (BAP cluster) based on their genotypic profiles (ST, serotype, and *fimH* type) [53].

WGS SPAdes assemblies were screened for acquired AMR genes and point mutations in intrinsic AMR genes using ResFinder 4.1 (available at http://www.genomicepidemiology.org/services/ (accessed on 13 February 2021)) [54]. 

Class 1 integron sequences were extracted from WGS assemblies using Geneious 2020.2.1 (Biomatters Limited, Auckland, New Zealand), and submitted to GenBank under accession numbers ON087280 and ON087281. PlasmidFinder 2.1 was used to detect plasmids in WGS assemblies [55] (available at http://www.genomicepidemiology.org/services/ (accessed on 19 February 2022)). ST58 and ST963 IncF plasmid sequences were obtained by mapping raw sequence reads to the reference IncF plasmids pCERC4 (GenBank accession KU578032) and pCE2050_A (GenBank accession CP073622). ST58 and ST963 IncF plasmids were further characterised by their F-plasmid replicon sequence type (IncF RST) using pMLST 2.0 [55] (available at http://www.genomicepidemiology.org/services/ (accessed on 15 February 2022)). 

VFs were identified by screening isolate WGS SPAdes assemblies using VirulenceFinder 2.0 (available at http://www.genomicepidemiology.org/services/ (accessed on 6 March 2021)) [56] and ABRicate VFDB (https://github.com/tseemann/abricate (accessed on 6 March 2021)) [57] (available at https://usegalaxy.org.au/ (accessed on 6 March 2021)). Isolates were assessed for the presence of VFs associated with ExPEC (*n* = 27)—adhesins (*afa/dra*, *fimH*, *iha*, *papA/papC*, *sfa*/*foc*, *tsh*), invasins (*gimB*, *ibeA*), iron acquisition (*fyuA/irp/ybt*, *ireA*, *iroN*, *iutA/iucA*, *sitA*), protectins (*iss*, *neuC*, *traT*), toxins (*astA*, *clb*, *cnf1*, *hly*, *sat*, *usp*, *vat*), miscellaneous (*ompT*, *pic*, *malX*), and capsules (*kpsM* II) [10,58,59]—along with VFs associated with IPEC (*n* = 7), i.e., AAF, *afa*, *aggR*, *bfpA*, *eae*, *ehx*, and *stx* [9]. The presence of additional VFs—including bacteriocins (colicins and microcins), *chuA, kpsD/E*, *lpfA*, and *senB*—was also assessed. 

VF profiles were analysed to determine whether the isolates exhibited an ExPEC pathotype. Five of the twenty-seven ExPEC-associated VFs were considered to be key VFs (*afa/dra*, *iutA*, *kpsM* II, *papA/papC*, and *sfa*/*foc*) [58]. Isolates harbouring ≥2 of these 5 key ExPEC VFs were defined as ExPEC [58]. Isolates carrying <2 of the 5 key ExPEC VFs were defined as follows: “ExPEC-potential” if they harboured ≥5 of 27 ExPEC-associated VFs, “ExPEC-like” if they harboured <5 ExPEC-associated VFs but ≥5 total VFs (ExPEC-associated and additional VFs), or “low pathogenicity” if they carried <5 total VFs [19]. 

### 2.6. Phenotypic Antimicrobial Susceptibility Testing of E. coli Strains

The 12 representative isolates selected for WGS—i.e., one isolate for each of the 12 *E. coli* strain types—underwent additional phenotypic antimicrobial susceptibility testing against a panel of 15 antibiotics representing 11 antimicrobial categories (Supplementary Table S3), using the European Committee on Antimicrobial Susceptibility Testing (EUCAST) criteria [60]. Isolates were evaluated as susceptible or resistant using the EUCAST breakpoint criteria (v 9.0 available at http://www.eucast.org/clinical_breakpoints/ (accessed on 22 October 2019)), or the Clinical and Laboratory Standards Institute (CSLI) breakpoint criteria (CLSI M100 ED29:2019 available at https://clsi.org/standards/products/free-resources/access-our-free-resources/ (accessed on 22 October 2019)) when EUCAST breakpoints were unavailable. A GHFF negative control isolate—*E. coli* FF1170 (EnteroBase Barcode ESC_JA9915AA)—was included as a quality control and to assist in evaluating zone diameters, as previously described [19]. Where breakpoint data were unavailable, resistance was defined as growth up to the edge of the disk, and intermediate resistance was evaluated as inhibition zone diameters smaller than the negative control isolate FF1170, but not to the edge of the disc. Multidrug resistance was defined as acquired phenotypic resistance to at least one antimicrobial in three or more categories [61].

### 2.7. Phylogenetic Analysis of E. coli Strains

Isolates phylogenetically related to the 12 GHFF *E. coli* strains were identified in EnteroBase by searching sequence types according to the Achtman seven-gene MLST scheme (available at https://enterobase.warwick.ac.uk/species/index/ecoli (accessed on 15 February 2022)) [52]. Phylogenetic comparison of EnteroBase and GHFF isolates was performed in EnteroBase using GrapeTree to construct rapid neighbour joining (RapidNJ) minimum spanning trees based on the core genome’s multilocus sequence typing (cgMLST) V1 + hierarchical clustering (HierCC) V1 scheme [62]. Trees containing >500 isolates were refined, with all resulting GrapeTrees containing between 27 and 439 isolates. GitHub URL links for interactive versions of all GrapeTree cgMLST phylogenetic trees are provided in Supplementary Table S4. 

GrapeTree clusters containing the ST58 O8:H25 and ST963 onon-typable:H18 GHFF strains were used to construct maximum likelihood trees [52] based on the RAxML of non-repetitive core SNPs (minimum presence 95%), using the EnteroBase SNP Project dendrogram module against an appropriate reference genome (ST58 EnteroBase Barcode ESC_QA8493AA and ST963 EnteroBase Barcode ESC_LA0140AA) [52]. Metadata associated with cluster isolates were obtained from EnteroBase (https://enterobase.warwick.ac.uk/species/index/ecoli (accessed on 15 February 2022)) and associated NCBI BioSample links (https://www.ncbi.nlm.nih.gov/biosample (accessed on 15 February 2022)).

## 3. Results

### 3.1. GHFF Pup Metadata

GHFF pups (*n* = 53) ranged in estimated age from 4 days to 15 weeks (mean age, 75 days) at entry to care. Pups entering care in November or December 2018 were either found alone on the ground (*n* = 11) or with their deceased mother (*n* = 2), and ranged in age from 4 to 56 days (mean age = 30.2 days). Pups entering care on 24 January 2019 (*n* = 40) were affected by a heat stress event, and ranged in age from 76 to 105 days (mean age = 89.5 days). Of the 53 pups entering care, 15 were housed in small groups with one of four wildlife carers, and 38 pups (all heat-stress-affected) were co-housed in a single large outdoor aviary, as they were old enough to self-feed on provided fruit and water. Two pups were administered prophylactic antibiotics (amoxicillin plus clavulanic acid), one pup received topical antimicrobial ointment to treat wing burns, and the forty heat-stressed pups received subcutaneous fluids to treat dehydration. Sample collection data and associated metadata for individual GHFF pups are available in Supplementary Data S1.

### 3.2. Selective Culture of Amoxicillin-Resistant E. coli from GHFF Pups

**Figure 1:**
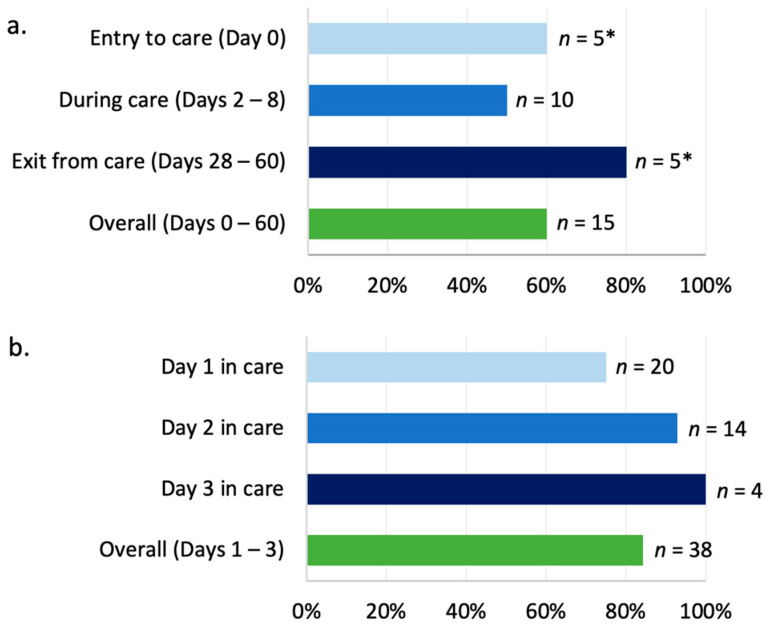
Prevalence of amoxicillin-resistant *Escherichia coli* in detected in faecal samples from orphaned, abandoned, and heat-stress-affected grey-headed flying-fox (GHFF) pups entering care in South Australia during the 2018/2019 pup season. The *y*-axis indicates when pups were sampled relative to entering care. The number (*n*) of pup faecal samples tested is listed adjacent bars. (**a**) Pups housed alone or in small groups; prevalence of amoxicillin-resistant *E. coli* in GHFF pups at entry to care, during care, and at exit from care. Asterisks (*) indicate that the same five pups were sampled both at entry to care and at exit from care. (**b**) Pups co-housed in one large group; prevalence of amoxicillin-resistant *E. coli* in GHFF pups 1–3 days after entering care.

Amoxicillin-resistant *E. coli* were cultured from 41 of the 53 (77.4%) GHFF pups sampled (Supplementary Data S1). For GHFF pups cared for in small groups, the overall prevalence of amoxicillin-resistant *E. coli* was lower (60%, 9 of 15) (Figure 1a) compared with pups co-housed in one large group (84.2%, 32 of 38) (Figure 1b). 

Of the five pups that were sampled at both entry to care (day 0) and just prior to exit from care (day 28–60), amoxicillin-resistant *E. coli* were detected in three pups (60%) at entry and in four pups (80%) at exit (Figure 1a). For the GHFF pups co-housed in one large group (*n* = 38), the prevalence of amoxicillin-resistant *E. coli* was lowest in pups sampled after one day in care (75%, 15 of 20), and highest in pups sampled after three days in care (100%, 4 of 4) (Figure 1b).

### 3.3. Strain Typing of Amoxicillin-Resistant E. coli Isolates

A total of 49 amoxicillin-resistant *E. coli* isolates were cultured from 58 faecal samples that were collected from 53 individual GHFF pups (Supplementary Data S1). PCR phylotyping of the 49 isolates identified four different phylogroup pattern types, namely, A, A/C, B1, and D/E (Supplementary Table S1). Nineteen isolates were class 1 integron integrase (*intI1*)-positive and harboured one of two integron types based on their gene cassette array patterns identified in HS458/JL-D2 PCRs (Supplementary Table S1). The genome fingerprints (ERIC and CGG PCRs) were correlated with phylogroup PCR patterns and integron status, and all 49 isolates were assigned to one of 12 distinct *E. coli* strain types (Supplementary Table S1). 

### 3.4. Whole-Genome Sequencing of Amoxicillin-Resistant E. coli Strains

One representative isolate from each of the 12 distinct strain types identified was selected to undergo whole-genome sequencing (WGS) (Supplementary Table S1). WGS identified 10 different sequence types (STs), with three strains belonging to the ST10 clonal complex (ST10 and ST48) and two strains to ST58 (Table 1). The 12 strains all belonged to different serotypes, with 11 H-antigen types and seven O-antigen types identified, and four strains were O-non-typable (ONT) (Table 1). Phylotyping of WGS assemblies in EnteroBase, using ClermonTyping, determined that the 12 strains belonged to three phylogroups: A (*n* = 6), B1 (*n* = 5), and D (*n* = 1) (Table 1). 

### 3.5. Phenotypic Antimicrobial Resistance Profiles of Amoxicillin-Resistant E. coli Strains

Antimicrobial susceptibility testing of the 12 representative isolates selected for WGS against a panel of 15 antibiotics (Supplementary Table S3) identified additional phenotypic resistance to a total of 10 antibiotics from 8 antimicrobial categories, across the 12 *E. coli* strain types (Supplementary Table S5). Two of the twelve strains—ST963 and ST2144—also exhibited resistance to amoxicillin plus clavulanic acid, and ST963 was also resistant to first- and third-generation cephalosporins (Table 1, Supplementary Table S5). Seven strains were resistant to tetracycline, three strains showed resistance to one aminoglycoside (spectinomycin or streptomycin), two strains were resistant to trimethoprim/sulfamethoxazole, and one strain was resistant to chloramphenicol (Table 1, Supplementary Table S5). Five of the twelve *E. coli* strains were classified as multidrug resistant, exhibiting resistance to three or four antimicrobial categories (Table 1, Supplementary Table S5). None of the 12 *E. coli* strains exhibited resistance to fluoroquinolones or carbapenems (Supplementary Table S5).

### 3.6. Genetic Antimicrobial Resistance Determinants of E. coli Strains

In 11 of the 12 *E. coli* strain types, amoxicillin resistance was conferred by one of three *bla*_TEM_ gene variants—*bla*_TEM-1B_ (*n* = 9), *bla*_TEM-1C_ (*n* = 1), or *bla*_TEM-33_ (*n* = 1)—and the 12th strain (ST963) harboured a chromosomally located *bla*_CMY-2_ gene (Table 1). The *bla*_TEM-33_ and *bla*_CMY-2_ genes also conferred resistance to amoxicillin plus clavulanic acid, and *bla*_CMY-2_ also conferred resistance to first- and third-generation cephalosporins (Table 1).

Two *E. coli* strains (ST48 O4:H26 and ST963 ONT:H18) carried class 1 integrons with the IS*26* transposase alternate 3′ sequence—specifically, *intI1-dfrA17-aadA5-*IS*26* and *intI1-dfrA5-*IS*26*, respectively. The *dfrA17* and *dfrA5* genes conferred resistance to trimethoprim, and both strains co-harboured a *sul2* gene that conferred resistance to sulphonamides (Table 1). The integron-associated *aadA5* gene conferred intermediate resistance to spectinomycin, and the combination of *aph(3″)-Ib* and *aph(6)-Id* conferred resistance to streptomycin, in two strains (Table 1). Two tetracycline-resistance genes were identified—*tet*(A) (*n* = 5) and *tet*(B) (*n* = 2)—along with a single chloramphenicol-resistance gene, *catA1* (Table 1).

### 3.7. Virulence Profiles of E. coli Strains

WGS analysis identified a total of 12 ExPEC-associated virulence factors (VFs) from the 12 *E. coli* strains, with each strain carrying between one and eight ExPEC VFs (Table 1). The most frequent VF in the 12 *E. coli* strains was *fimH* (*n* = 11, 91.7%), followed by *ompT* (*n* = 7, 58.3%), *iss* (*n* = 6, 50.0%), and *hlyF* (*n* = 4, 33.3%) (Table 1). Three VFs associated with iron acquisition were identified in *E. coli* strains: aerobactin (*iutA*/*iucA*) (*n* = 3, 25.0%), yersiniabactin (*fyuA/irp/ybt*) (*n* = 2, 16.7%), and *iroN* (*n* = 2, 16.7%) (Table 1). Bacteriocins (i.e., colicins and/or microcins) were present in five (41.7%) strains (Table 1). No IPEC-specific VFs were identified in any of the 12 strains. 

Diverse plasmid replicons were identified in all 12 strains, with between one and four carried by each strain (Table 1). The two ST58 strains, serotypes O8:H25 and ONT:H37, carried the IncF RST F2:A-:B1 and IncF RST F18:A-:B1 plasmids, respectively, which harboured multiple VFs, including aerobactin, colicin, *iroN*, and *traT* (Table 1). The ST963 ONT:H18 strain carried an IncF RST F29:A-:B10 plasmid (Table 1).

None of the 12 *E. coli* strains met the criteria for ExPEC; however, 4 strains each carried one key ExPEC VF (Table 1). The 12 strains were assigned to one of three pathotypes: ExPEC-potential (*n* = 5, 41.7%), ExPEC-like (*n* = 1, 8.3%), and low pathogenicity (*n* = 6, 50.0%) (Table 1). 

### 3.8. Prevalence of E. coli Strain Types in GHFF Pups

**Figure 2:**
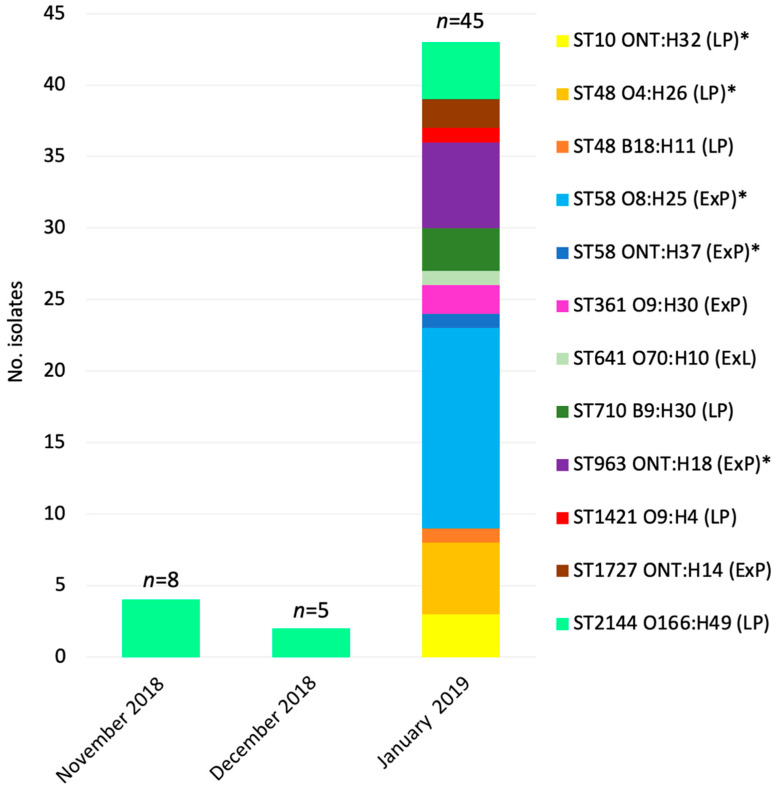
Amoxicillin-resistant *E. coli* strain types detected in faecal samples from orphaned, abandoned, and heat-stress-affected grey-headed flying fox pups entering care in South Australia during the 2018/2019 pup season. Numbers (*n*) above the bars indicate the number of faecal samples cultured. ExP, ExPEC-potential; ExL, ExPEC-like; LP, low pathogenicity. Asterisks (*) indicate multidrug-resistant strains.

Strain typing of all 49 *E. coli* isolates and WGS of the 12 representative isolates determined that the most frequent strain type in the 53 pups was the ST58 serotype O8:H25 (*n* = 14 pups), followed by ST2144 O166:H49 (*n* = 10 pups), ST963 ONT:H18 (*n* = 6 pups), and ST48 O4:H26 (*n* = 5 pups) (Figure 2). The majority of pups carried a single amoxicillin-resistant strain (*n* = 37/53), and four pups carried multiple strains (Supplementary Data S1).

All GHFF pups sampled in November or December 2018, and resampled in January 2019, carried only the *E. coli* strain ST2144 O166:H49 (*n* = 10), which was not detected in any of the 40 heat-stressed pups entering care on 24 January 2019 (Figure 2). The 40 heat-stressed pups sampled in late January 2019 carried 11 different amoxicillin-resistant *E. coli* strains (Figure 2, Supplementary Data S1).

The six *E. coli* strains with ExPEC characteristics accounted for 53.1% of isolates (26 of 49), and low-pathogenicity strains accounted for 46.9% of the isolates (23 of 49) from GHFF pups (Figure 2, Supplementary Data S1). Notably, three strains (ST58 O8:H25, ST58 ONT:H37, and ST963 ONT:H18) that accounted for 42.9% of all isolates (21 of 49) were defined as both ExPEC-potential pathotypes and multidrug resistant (Figure 2, Supplementary Data S1).

### 3.9. Prevalence of Antimicrobial Resistance in GHFF Pups

The prevalence of amoxicillin-, amoxicillin-plus-clavulanic-acid-, and cephalosporin-resistant *E. coli* in the 53 GHFF pups was 77.4% (*n* = 41 pups), 24.5% (*n* = 13 pups), and 11.3% (*n* = 6 pups), respectively (Table 1, Supplementary Data S1). Amoxicillin-resistant *E. coli* that co-harboured resistance to aminoglycosides, trimethoprim plus sulphonamide, tetracyclines, and chloramphenicol were present in 37.7% (*n* = 20), 35.8% (*n* = 19), 26.4% (*n* = 14), and 1.9% (*n* = 1) of GHFF pups, respectively, and overall, 50.9% of GHFF pups (*n* = 27) carried multidrug-resistant *E. coli* strains (Table 1, Supplementary Data S1).

GHFF pups entering care in November or December 2018 all carried *E. coli* exhibiting resistance to two antibiotic categories (amoxicillin and amoxicillin plus clavulanic acid), whereas the heat-stressed pups entering care in late January carried *E. coli* exhibiting resistance to eight antimicrobial categories.

### 3.10. Phylogenetic Analysis of E. coli Strains with ExPEC Characteristics

GrapeTree phylogenetic analysis of GHFF *E. coli* strains with ExPEC characteristics (*n* = 6 strains), as well as closely related isolates identified in EnteroBase, placed two strains (ST963 ONT:H18 and ST361 O9:H30) in lineages predominantly composed of human-sourced isolates, and four strains (ST58 O8:H25, ST58 ONT:H37, ST641 O70:H10, and ST1727 ONT:H14) in lineages composed of isolates sourced from animals and humans. All GrapeTree phylogenetic lineages containing the GHFF strains with ExPEC characteristics also contained human- and/or animal-sourced ExPEC isolates.

**Figure 3:**
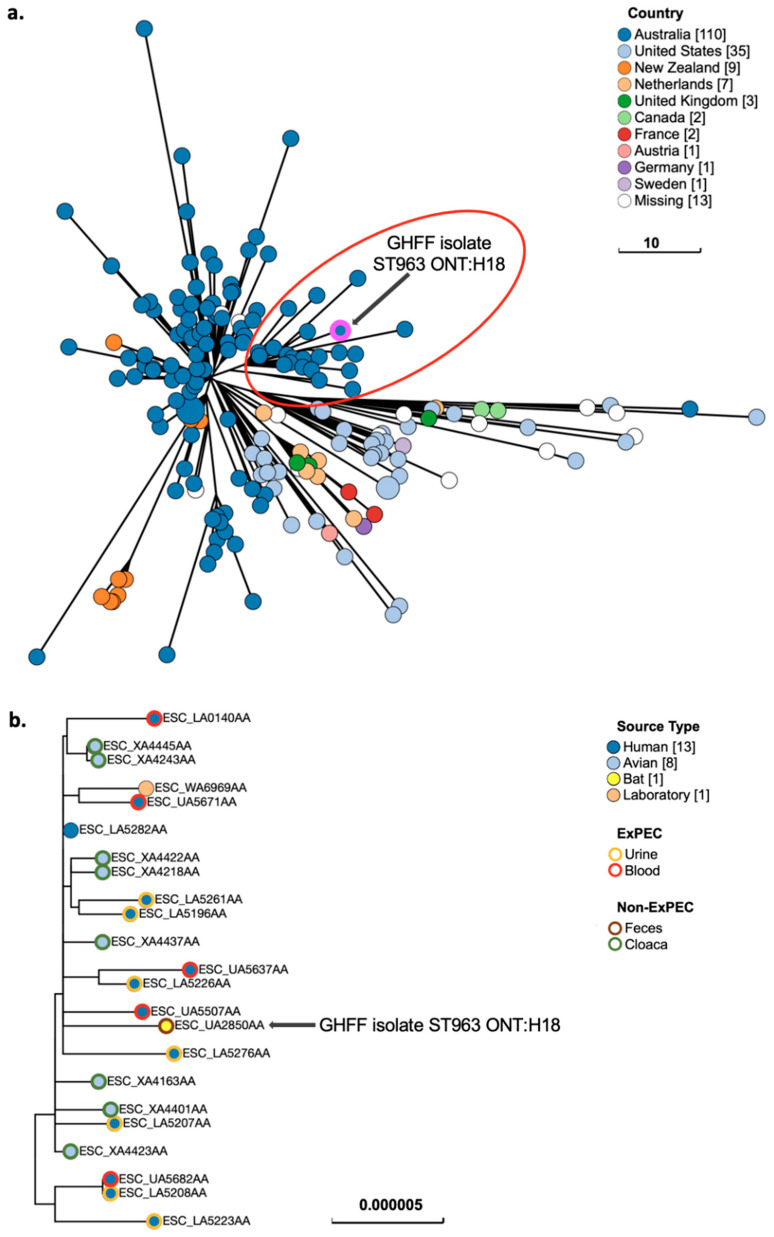
Phylogenetic and metadata analysis of *E. coli* ST963 ONT:H18 from grey-headed flying fox (GHFF) pups and closely related isolates from human and animal sources identified in EnteroBase. (**a**) GrapeTree phylogeny constructed using a rapid neighbour joining (RapidNJ) minimum spanning tree based on the cgMLST V1 + hierarchical clustering (HierCC) V1 scheme. Clusters containing GHFF isolates are circled in red. Scale bars indicate the number of cgMLST allelic differences. GitHub URL links for interactive versions of all GrapeTrees are provided in Supplementary Table S4. (**b**) Core genome SNP analysis and associated metadata tables of GrapeTree clusters containing GHFF pup isolates. Maximum likelihood trees were based on the RAxML of non-repetitive core SNPs using the EnteroBase SNP Project dendrogram module against the reference genome ESC_LA0140AA. Isolate ID indicates EnteroBase Barcode. Scale bars indicate the number of substitutions per site.

Phylogenetic analysis of ST963 ONT:H18 isolates (*n* = 184) placed the GHFF strain within an Australian-sourced cluster (Figure 3a). The Australian cluster contained ST963 ONT:H18 isolates predominantly cultured from human blood or urine (*n* = 11) and wild silver gull cloacal swabs (*Chroicocephalus novaehollandiae*) (*n* = 8) (Figure 3b). SNP tree analysis of the cluster determined that the GHFF ST963 ONT:H18 strain was most closely related to four human-sourced ExPEC isolates from blood (*n* = 2) and urine (*n* = 2) (Figure 3b). 

**Figure 4:**
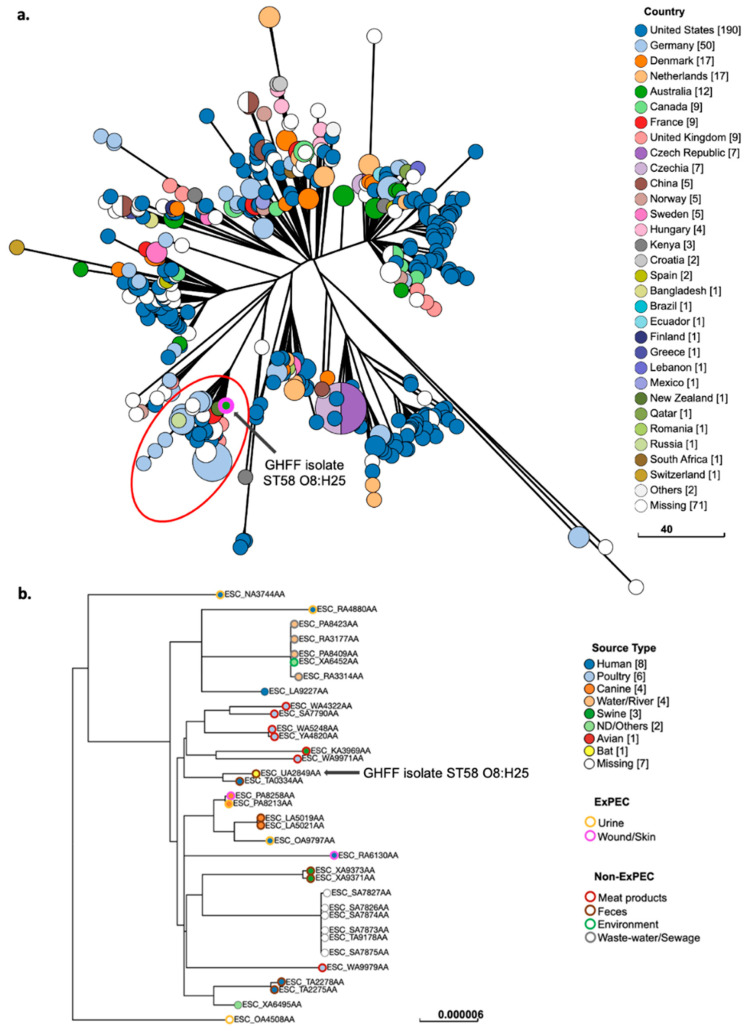
Phylogenetic and metadata analysis of *E. coli* ST58 O8:H25 from grey-headed flying fox (GHFF) pups and closely related isolates from human, animal, and environmental sources identified in EnteroBase. (**a**) GrapeTree phylogeny constructed using a rapid neighbour joining (RapidNJ) minimum spanning tree based on the cgMLST V1 + hierarchical clustering (HierCC) V1 scheme. Clusters containing GHFF isolates are circled in red. Scale bars indicate the number of cgMLST allelic differences. GitHub URL links for interactive versions of all GrapeTrees are provided in Supplementary Table S4. (**b**) Core genome SNP analysis and associated metadata tables of GrapeTree clusters containing GHFF pup isolates. Maximum likelihood trees were based on the RAxML of non-repetitive core SNPs using the EnteroBase SNP Project dendrogram module against the reference genome ESC_QA8493AA (removed from the SNP tree image for clarity). Isolate ID indicates EnteroBase Barcode. Scale bars indicate the number of substitutions per site.

Analysis of ST58 O8:H25 isolates (*n* = 439) showed that the isolates were sourced from diverse countries and were not clustered according to geographical location (Figure 4a). The GHFF ST58 O8:H25 *fimH34* strain was the only Australian-sourced isolate in a large cluster (*n* = 35) belonging to the ST58 BAP2 sub-lineage (Figure 4a). The GHFF cluster contained isolates from diverse host species and source types, including human ExPEC (*n* = 4), canine ExPEC (*n* = 2), poultry and swine meat products (*n* = 7), and faeces/sewage (*n* = 12) (Figure 4b). In SNP tree analysis of the cluster, the GHFF ST58 O8:H25 strain was most closely related to an isolate cultured from human faeces in New Zealand (Figure 4b).

**Figure 5:**
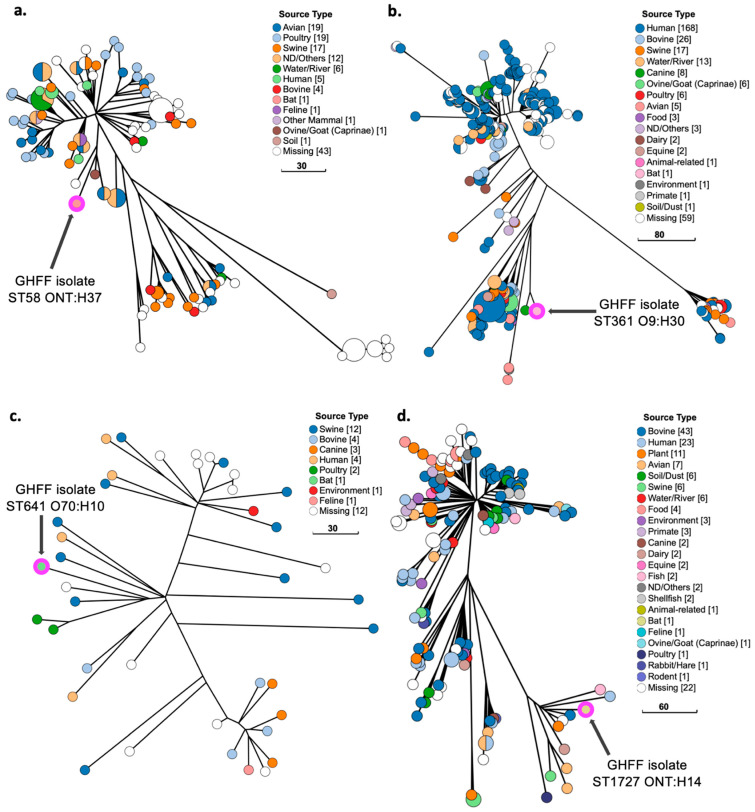
Phylogenetic and metadata analysis of four *E. coli* isolates with ExPEC characteristics from grey-headed flying fox (GHFF) pups and closely related isolates from human, animal, and environmental sources identified in EnteroBase. GrapeTree phylogeny constructed using a rapid neighbour joining (RapidNJ) minimum spanning tree based on the cgMLST V1 + hierarchical clustering (HierCC) V1 scheme. Scale bars indicate the number of cgMLST allelic differences. GitHub URL links for interactive versions of all GrapeTrees are provided in Supplementary Table S4. (**a**) ST58 ONT:H37; (**b**) ST361 O9:H30; (**c**) ST641 O70:H10; (**d**) ST1727 ONT:H14. All ST1727 H49 isolates (a divergent cluster), and two isolates with branch lengths > 200 cgMLST allelic differences, were excluded for clarity.

The single ST58 ONT:H37 *fimH32* GHFF isolate belonged to an ST58 ONT:H37 BAP4 sub-lineage (*n* = 130) composed predominantly of poultry-, wild-bird-, and livestock-sourced isolates, plus five human-associated isolates (Figure 5a). The ST58 sub-lineage contained numerous APEC; however, the GHFF ST58 ONT:H37 isolate belonged to a small cluster containing isolates from one wild bird and goat faeces (Figure 5a). 

Phylogenetic analysis of ST361 O9:H30 isolates (*n* = 323) determined that the majority were sourced from humans (*n* = 168), including ExPEC cultured from urine and blood, along with isolates associated with livestock and companion animals (Figure 5b). The GHFF ST361 O9:H30 strain belonged to a divergent lineage containing only two isolates, with the second being a canine ExPEC cultured from urine collected from a dog in Melbourne, Australia (Figure 5b).

Analysis of ST641 O70:H10 isolates (*n* = 40) found that they were from diverse sources, including livestock, humans, and companion animals (Figure 5c). Numerous isolates were ExPEC, and were cultured from human blood and urine, canine urine and lungs, and bovine mastitis (Figure 5c). The ST641 O70:H10 isolate was clustered with isolates sourced from human blood (*n* = 1), swine faeces and meat (*n* = 3), and poultry (*n* = 2) (Figure 5c).

The ST1727 phylogenetic tree (*n* = 153) was composed of isolates sourced from diverse animals, humans, and food products, and included ExPEC cultured from human and canine urine (Figure 5d). The GHFF ST1727 ONT:H14 strain belonged to a small cluster (*n* = 11) of ST1727 O-variable:H14 isolates from diverse sources, including dairy, meat, fish, and plant products, as well as human faeces (Figure 5d).

### 3.11. Phylogenetic Analysis of E. coli Strains with Low Pathogenicity

GrapeTree phylogenetic analysis of GHFF isolates with low pathogenicity characteristics (*n* = 6), along with closely related isolates identified in EnteroBase, placed all strains in lineages comprising isolates sourced from animals and humans. GrapeTree isolates were from diverse sources, including faeces, meat and dairy products, the environment, and occasional ExPEC origins. Three of six low-pathogenicity GHFF isolates belonged to the ST10 complex, namely, ST10 ONT:H32, ST48 O4:H26, and ST48 B18:H11. The three remaining low-pathogenicity GHFF isolates (ST710 B9:H30, ST1421 O9:H4, and ST2144 O166:H49) belonged to phylogenetically distinct lineages.

**Figure 6:**
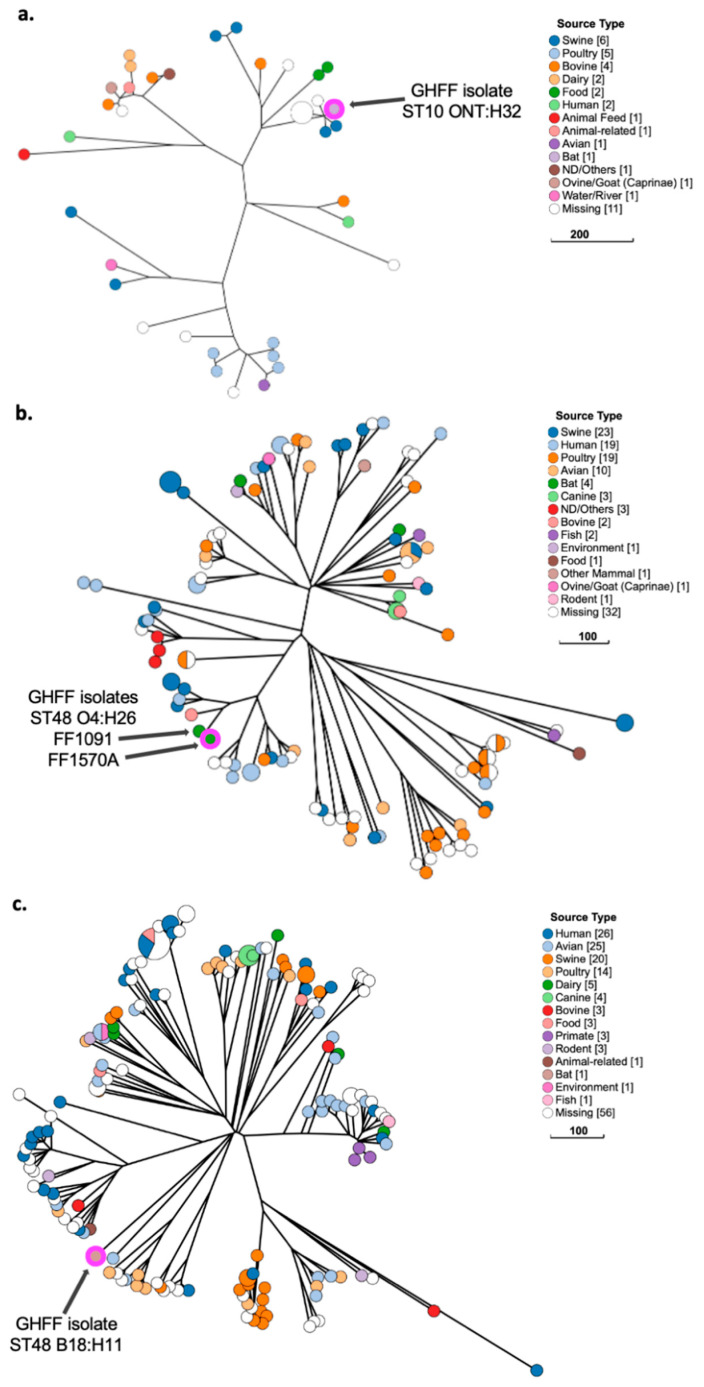
Phylogenetic and metadata analysis of three low-pathogenicity ST10 complex *E. coli* isolates from grey-headed flying fox (GHFF) pups and closely related isolates from human, animal, and environmental sources identified in EnteroBase. GrapeTree phylogeny constructed using a rapid neighbour joining (RapidNJ) minimum spanning tree based on the cgMLST V1 + hierarchical clustering (HierCC) V1 scheme. Scale bars indicate the number of cgMLST allelic differences. GitHub URL links for interactive versions of all GrapeTrees are provided in Supplementary Table S4. (**a**) ST10 ONT:H32; (**b**) ST48 O4:H26; (**c**) ST48 B18:H11.

The ST10 ONT:H32 isolate was clustered with isolates sourced from swine (faeces and meat), bovine, and food products, and was most closely related to two swine-associated isolates from unknown sample types (Figure 6a). ST48 O4:H26 belonged to a cluster of isolates sourced from bovine milk (*n* = 1), swine faeces (*n* = 4), and human faeces (*n* = 1); however, it was almost identical (four cgMLST allelic differences) to the isolate FF1091, which was sourced from an adult GHFF at the same rehabilitation facility in February 2018 (Figure 6b). The ST48 B18:H11 isolate was placed in a broad cluster that predominantly contained isolates sourced from domestic poultry (e.g., geese, chickens, turkeys), but also contained two enterotoxigenic *E. coli* (ETEC) (Figure 6c). 

**Figure 7:**
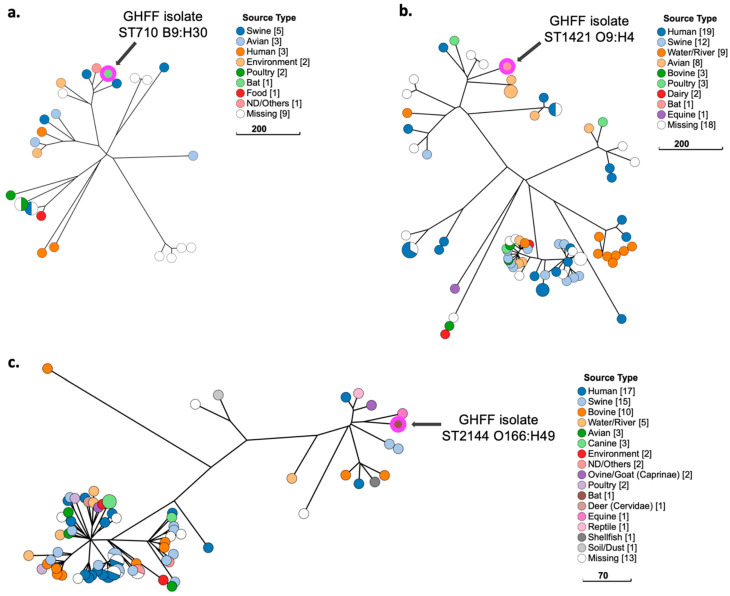
Phylogenetic and metadata analysis of three low-pathogenicity *E. coli* isolates from grey-headed flying fox (GHFF) pups and closely related isolates from human, animal, and environmental sources identified in EnteroBase. GrapeTree phylogeny constructed using a rapid neighbour joining (RapidNJ) minimum spanning tree based on the cgMLST V1 + hierarchical clustering (HierCC) V1 scheme. Scale bars indicate the number of cgMLST allelic differences. GitHub URL links for interactive versions of all GrapeTrees are provided in Supplementary Table S4. (**a**) ST710 B9:H30; (**b**) ST1421 O9:H4; (**c**) ST2144 O166:H49.

ST710 B9:H30 belonged to a cluster of four isolates, of which the GHFF isolate was the only non-Shiga-toxin-producing *E. coli* (non-STEC) (Figure 7a). The three STEC were sourced from swine (*n* = 2) and the environment (*n* = 1), and all carried the *stx2* virulence gene, which was absent in the GHFF isolate (Figure 7a). The ST1421 O9:H4 isolate was clustered with isolates sourced from wild gull faeces (*n* = 3), poultry (*n* = 1), and human faeces (*n* = 1) (Figure 7b). ST2144 O166:H49 was placed in a cluster with isolates from diverse sources—including human faeces (*n* = 2), goat faeces (*n* = 1), swine (*n* = 2), bovines (*n* = 2), oysters (*n* = 1), and a reptile (*n* = 1)—and was most closely related to an equine ExPEC sourced from a uterine swab (Figure 7c).

## 4. Discussion

This study determined that the prevalence of amoxicillin-resistant *E. coli* in GHFF pups was extremely high (77.4%), and was not consistent with the low levels previously detected in adult GHFFs from the ABP colony (4.1%) [19]. Additionally, 24.5% of pups carried *E. coli* strains that were also resistant to amoxicillin plus clavulanic acid, and 11.3% carried *E. coli* resistant to first- and third-generation cephalosporins, neither of which have been detected in *E. coli* from adults in the ABP colony [19]. Amoxicillin, amoxicillin plus clavulanic acid, and 1st–3rd generation cephalosporins are considered “veterinary critically important antimicrobial agents”, due to the wide variety of diseases they treat in a broad range of animal species [4]. Overall, 50.9% GHFF pups carried multidrug-resistant *E. coli* strains, in contrast to only 0.81% of adults from the ABP colony [19]. The amoxicillin-resistant *E. coli* strains from GHFF pups did not exhibit resistance to fluoroquinolones, fourth- and fifth-generation cephalosporins, or carbapenems, which are deemed critically important antimicrobials for human and veterinary medicine [3,4].

The high prevalence of amoxicillin-resistant *E. coli* in pups sampled at entry to care (60%) and within 24 h of entering care (75%) indicates that they acquired resistant *E. coli* prior to entering care. The increase in prevalence of *E. coli* ST2144 observed in five co-housed pups between entry to care and exit from care most likely occurred via horizontal transmission between GHFF pups. This finding is consistent with reports on adult GHFFs that found that the occurrence of beta-lactam-resistant *E. coli* and class 1 integrons was higher in captive adults (6.5% and 41.2%, respectively) compared to wild adults (3.5% and 5.3%, respectively) [19,45].

Several factors are known to influence the gut microbiota composition in young mammals of different species, and these may have contributed to the high prevalence of amoxicillin-resistant *E. coli* observed in GHFF pups. Firstly, *E. coli* is typically one of the first bacterial species to colonise the gut in newborn mammals [63], including humans [64], resulting in a higher prevalence and abundance of *E. coli* in babies compared to adults [18,64,65,66]. Secondly, a higher prevalence of antimicrobial-resistant *E. coli* has been observed in the gut microbiota of pre-weaned young compared to adults—including in human infants [65,67], calves [68,69], and piglets [70]—and is mostly likely attributable to the greater abundance of *E. coli* in pre-weaned young compared to adults [65]. Thirdly, the loss and reacquisition of antimicrobial-resistant *E. coli* over time has been observed in co-housed young animals—for example, in calves, where antimicrobial-resistant *E. coli* are most likely lost as the gut microbiome matures, and then reacquired via horizontal transmission from other calves or from new environmental sources [69,71].

Environmental contamination with human and animal effluent, wastewater, stormwater, and agricultural runoff has been suggested as possible acquisition sources of antimicrobial-resistant bacteria for wildlife [29]. As flying foxes are either in flight or arboreal, they display a “dipping” behaviour, which involves skimming across the surface of a freshwater body during flight to wet their belly fur and acquire drinking water. Dipping can potentially expose a flying fox, and the young pup clinging to its mother during the first month of life [42], to antimicrobial-resistant bacteria in water sources, when the wet fur is licked or when a pup suckles milk from its mother. Although wild GHFF pups begin to fly short distances and forage for food from about 3 months of age, they are not fully weaned until approximately 4–5 months old [42,72]. Female GHFFs form maternity roosts within a colony, where pups are left in crèche groups from approximately one month old while their mothers forage at night [42]. This close roosting behaviour of pups in crèches is also likely to facilitate the horizontal transmission of antimicrobial-resistant faecal bacteria between pups [34,73].

In pups that entered care during November and December 2018 (*n* = 13), only one strain of amoxicillin-resistant *E. coli* was detected (ST2144). In contrast, the GHFF pups that entered care on 24 January 2019 following a heat-stress event (*n* = 40) carried a high diversity of amoxicillin-resistant *E. coli*, with 11 distinct strains identified. These findings suggest that the *E. coli* strains present in the January heat-stressed pups were acquired from different environmental sources, and/or there was an increase in faecal bacterial contamination of environmental sources in January. During November and December 2018, the reported monthly rainfall totals in Adelaide were 36 mm and 20 mm, respectively; however, between 20 December 2018 and 24 January 2019, zero rainfall occurred [74]. Additionally, a three-day-long heatwave occurred in January 2019, with maximum temperatures reaching 38.9˚C and 40.9 °C on 22 and 23 January 2019, respectively, and 46.6 °C on 24 January, when the heat-stress event occurred. These factors may have contributed to increased *E. coli* contamination in Adelaide water bodies utilised by GHFFs [75]. During heatwaves, nursing female GHFFs may preferentially utilise water and food sources that are located close to the colony, potentially exposing them to sources with increased levels of *E. coli* contamination [76].

In a study utilising Global Positioning System (GPS) telemetry to track the flight movements of adult GHFFs from the ABP colony, they were observed to visit several water sources, and presumably dipping to obtain drinking water [40]. These water sources included a lake in the Adelaide Botanic Gardens, and the River Torrens—both in close proximity to the GHFF colony—along with suburban drainage ponds and a quarry dam [40]. The River Torrens has been reported to have poor water quality, with faecal contamination identified in multiple reports [77,78,79]; thus, it is a potential source of amoxicillin-resistant *E. coli* for the ABP GHFF colony. 

An additional consideration is that wildlife entering captivity—even for short periods of time—may acquire antimicrobial-resistant *E. coli* whilst in care, either directly from their human carers, or indirectly from environmental contamination by humans, or from other wildlife and domestic animals in close proximity [22,30,80]. However, given that 10 amoxicillin-resistant *E. coli* strains were detected in GHFF pups within the first 24 h of entering care, it would be unlikely that these were all acquired from the captive environment. Additionally, the same *E. coli* strains were detected in pups taken into care at different times and/or at different locations, further suggesting acquisition from wild environmental sources prior to entering care.

The prevalence and abundance of antimicrobial-resistant *E. coli* in mammals is reported to decrease with age [65,69]; however, it is currently unknown whether the amoxicillin-resistant *E. coli* identified in pups’ gut microbiota persist into adulthood. Notably, carriage of amoxicillin-resistant *E. coli* was maintained throughout the time in care (up to 60 days) in four GHFF pups. The tendency for bats to aggregate may facilitate horizontal transmission and maintain the long-term circulation of antimicrobial-resistant *E. coli* strains within a co-housed group or colony [34,73]. The *E. coli* ST48 O4:H26 strain detected in five GHFF pups was almost identical to an isolate detected in an adult GHFF from the ABP colony 11.5 months earlier [19]. Notably, both the pup and adult were recovering from heat stress and co-housed in the same rehabilitation facility, suggesting that the *E. coli* ST48 O4:H26 strain acquired by adults and pups originated from the same source, and has been circulating for some time in this GHFF population. 

Phylogenetic analysis indicated that all 12 amoxicillin-resistant *E. coli* strains detected in GHFF pups originated from anthropogenic sources, with six strains known to cause extraintestinal infections in humans and/or animals [10]. Of particular concern was the detection of the human-associated ExPEC strain ST963 ONT:H18 in six GHFF pups, which also exhibited resistance to extended-spectrum beta-lactams. This GHFF strain belongs to an Australian *E. coli* ST963 clonal cluster, which is typically characterised by a chromosomal *bla*_CMY-2_ gene and an IncF F29:A-:B10 plasmid [81]. Australian ST963 clonal isolates have been widely detected in humans and silver gulls (*Chroicocephalus novaehollandiae*) across Australia [23,81], with genomic analysis indicating that interspecies transmission is occurring between humans and gulls [81]. Although phylogenetic analyses suggested that the GHFF pup ST693 strain was most closely related to Australian human-sourced isolates from blood and urine, rather than to gull-sourced isolates, it is likely that GHFFs are also part of the ST963 interspecies transmission pathways between humans and wildlife in Australia [81]. ST58 O8:H25 was the most frequently detected *E. coli* strain in the heat-stressed GHFF pups (*n* = 14). Although this ST58 BAP2 sub-lineage harbouring a ColV plasmid appears to have originated in poultry and swine, it is increasingly associated with blood and urinary tract infections in humans [53]. The high prevalence of amoxicillin-resistant *E. coli* strains with ExPEC characteristics in GHFF pups poses a significant health risk to pups, as these strains have the potential to cause pneumonia, as well as wound or blood infections [10]. These ExPEC strains—especially ST963 and ST58 O8:H25—also pose a significant zoonotic risk, and have the potential to cause extraintestinal infections in human carers [53,82]. 

Amoxicillin and amoxicillin plus clavulanic acid are probably the most commonly administered antibiotics to GHFF pups in care [43,83]. Given the high prevalence of resistance to these antibiotics in GHFF pups (77.4% and 24.5%, respectively), the need for adherence to antimicrobial stewardship guidelines, and only administering antibiotics when there is clinical infection, is paramount [84]. Two GHFF pups in this study received prophylactic doses of amoxicillin plus clavulanic acid upon entry to care, and both pups carried *E. coli* ST2144, which harbours a *bla*_TEM-33_ gene conferring resistance to amoxicillin plus clavulanic acid. In this scenario, there is the potential for selective pressure and risk of overgrowth of the resistant ST2144 strain in the gut microbiota, and although it was classified as having low pathogenicity, ST2144 does carry two significant virulence factors (*astA* and *lpfA*) that can contribute to extraintestinal disease [10]. Additionally, antimicrobial selection pressure, combined with other stressors—such as separation of the pup from its mother and changing the diet to artificial milk formula—creates increased potential for horizontal transfer of antimicrobial resistance genes and virulence genes between bacterial strains in the pups’ gut microbiome [85]. Consequently, horizontal gene transfer may drive the emergence of new strains of multidrug-resistant bacterial pathogens in the pups’ gut microbiome [84]. Given that heat-stress events and pup abandonments are likely to increase in the future due to climate change, there are likely to be increasing numbers of GHFF pups entering care each year [86,87], and many of these will require antimicrobial therapy. Administration of antibiotics to injured or sick GHFF pups carrying antimicrobial-resistant isolates may result in a poor response to treatment and a worsened prognosis for recovery and release back into the wild. 

Implementing biosafety measures for GHFFs in captive settings is important for pup health and human health. Husbandry recommendations to limit the horizontal transmission of resistant bacterial strains between co-housed pups in care, and to minimise the biological risks to both pups and their carers, include (i) individual housing of GHFF pups receiving antimicrobial therapy, (ii) reducing the number of pups in co-housing cages, (iii) preventing direct contact between pups from different co-housed groups, (iv) dedicated equipment for each pup cage, (v) frequent cleaning and disinfection of pup-associated equipment and cages, (vi) wearing gloves and changing them between cages/pups, plus frequent hand washing, and (vii) excluding contact between pups and other wildlife or domestic animals.

Further work is required to examine the transmission dynamics and persistence of antimicrobial-resistant bacteria in GHFF pups as they are transitioned from care to placement in larger groups in “crèche” cages, in preparation for release back into wild colonies when they reach juvenile age [43]. Of the thousands of GHFF pups entering care each year, approximately 75% will be successfully reared in captivity and released back into the wild [87], along with any antimicrobial-resistant bacteria that they carry, which then have the potential for dissemination to other animals, humans, and into the environment.

## Figures and Tables

**Table 1:** Genetic characterisation of amoxicillin-resistant strains from orphaned, abandoned, and heat-stress-affected grey-headed flying fox pups entering care in South Australia during the 2018/2019 pup season. Antimicrobial resistance (AMR) genes associated with class 1 integrons are highlighted in bold. Key ExPEC virulence factors are highlighted in bold. Multidrug resistance is defined as resistance to ≥3 antimicrobial categories (Cat.) and are highlighted in bold.

ST	Serotype	fimH Type	PG	AMR Genes (Corres-ponding Phenotypic Antibiotic *)	No. AMR Cat.	Virulence Factors	Pathotype #	Plasmids Identified
10	ONT:H32	fimH54	A	*aph(3″)-Ib* + *aph(6)-Id* (S-I)*, bla*TEM-1B (AML, AMP), *tet*(B) (TE)	**3**	*iss*	Low path.	IncR, IncX1
48	O4:H26	fimH23	A	***aadA5*** (SH-I), *bla*TEM-1B (AML, AMP), ***dfrA17*** *+ sul2* (W, SXT)*, tet*(A) (TE)	**4**	*-*	Low path.	p0111
48	B18:H11	fimH41	A	*bla*TEM-1B (AML, AMP), *sul3* (NT)	2	*ompT*	Low path.	p0111
58	O8:H25	fimH34	B1	*aph(3″)-Ib + aph(6)-Id* (S-R), *bla*TEM-1B (AML, AMP),***dfrA5** + sul2* (W, SXT)	**3**	*cia, cvaC, fyuA/irp/ybt, hlyF*, *iss, **iuc**/**iut***, *sitA, mchF, ompT, traT*	ExPEC-P.	ColRNAI, IncFIB + IncFII (IncF RST F2:A-:B1), IncQ1
58	ONT:H37	fimH32	B1	*bla*TEM-1B (AML, AMP), *catA1* (C)*, tet*(B) (TE)	**3**	*cba, cma, cvaC, hlyF*, *iroN*, *iss, **iuc**/**iut***, *sitA, lpfA, mchF, ompT, traT*	ExPEC-P.	ColRNAI, IncFIB + IncFIC(FII) (IncF RST F18:A-:B1), IncI1-I(Alpha)
361	O9:H30	fimH54	A	*bla*TEM-1C (AML, AMP), *tet*(A) (TE)	2	*astA, cia, cvaC, hlyF, iroN, iss, mchF, ompT*, *sitA*, *traT*	ExPEC-P.	IncFIB, IncFII
641	O70:H10	fimH25	B1	*bla*TEM-1B (AML, AMP),	1	*cia, iss*, *lpfA*, *ompT, **papC***	ExPEC-like	IncI1-I(Alpha)
710	B9:H30	fimH1582	A	*bla*TEM-1B (AML, AMP), *tet*(A) (TE)	2	*ompT*	Low Path.	IncFIA(HI1), IncFIB(K)
963	ONT:H18	fimH26	D	*bla*CMY-2 (AML, AMP, AMC, CL, CTX)	**3**	*air/eilA, chuA, fyuA/irp/ybt, kpsD/E*, *senB, sitA, traT*	ExPEC-P.	Col156 + IncFIB + IncFII(29)(IncF RST F29:A-:B10)
1421	O9:H4	Unknown	A	*bla*TEM-1B (AML, AMP), *tet*(A) (TE)	2	*ompT*, *sitA*	Low Path.	IncFIA(HI1), IncFIB(H89-PhagePlasmid)
1727	ONT:H14	fimH31	B1	*bla*TEM-1B (AML, AMP), *tet*(A) (TE)	2	*cvaC, hlyF, iroN, iss, **iuc**/**iut**, sitA, lpfA, mchF, traT, tsh*	ExPEC-P.	IncFIA, IncFIB, IncFII(FII)
2144	O166:H49	fimH87	B1	*bla*TEM-33 (AML, AMP, AMC)	2	*astA, lpfA*	Low Path.	Col156, IncI1-I(Alpha)

* Antibiotic abbreviations: AMP, ampicillin; AMC, amoxicillin + clavulanic acid; AML, amoxicillin; AMR Cat, antimicrobial resistance categories; C, chloramphenicol; CL, cephalexin; CTX, cefotaxime; I, intermediate resistance; NT, not tested; ONT, O-non-typable; PG, phylogroup; S, streptomycin; SH, spectinomycin; SXT, trimethoprim + sulfamethoxazole; W, trimethoprim. # Pathotype; Low Path, low pathogenicity; ExPEC-P, ExPEC potential.

## Data Availability

Whole-genome sequencing raw reads are available at the NCBI Sequence Read Archive (SRA) under BioProject ID PRJNA814458 and BioSample accession numbers SAMN26553891 to SAMN26553902; https://www.ncbi.nlm.nih.gov/bioproject/?term=PRJNA814458 (accessed on 10 March 2022). Assembled genome sequences are available at EnteroBase under Barcodes ESC_UA2845AA to ESC_UA2856AA; https://enterobase.warwick.ac.uk/species/index/ecoli (accessed on 13 February 2021). Class 1 integron sequences are available at NCBI GenBank under the accession numbers ON087280 and ON087281; https://www.ncbi.nlm.nih.gov/genbank/ (accessed on 29 March 2022). Supplementary Table S2 contains individual isolate accessions. GitHub URL links for interactive versions of all GrapeTree cgMLST phylogenetic trees are provided in Supplementary Table S4.

## Supplementary Materials

The following supporting information can be downloaded at: https://www.mdpi.com/article/10.3390/microorganisms10081589/s1, Supplementary Data S1: Individual GHFF pup data; Supplementary Table S1: Strain typing of amoxicillin-resistant *Escherichia coli* isolates; Supplementary Table S2: Individual EnteroBase Barcodes, along with BioSample and GenBank accession numbers; Supplementary Table S3: Antibiotic discs used for EUCAST susceptibility testing; Supplementary Table S4: URL links to interactive versions of GrapeTree cgMLST phylogeny trees; Supplementary Table S5: Phenotypic antimicrobial susceptibility testing of *E. coli* strains.

## References

[1] Poirel L., Madec J.Y., Lupo A., Schink A.K., Kieffer N., Nordmann P., Schwarz S. (2018). Antimicrobial resistance in *Escherichia coli*. Microbiol. Spectr..

[2] Guenther S., Ewers C., Wieler L.H. (2011). Extended-spectrum beta-lactamases producing *E. coli* in wildlife, yet another form of environmental pollution?. Front. Microbiol..

[3] WHO (2019). Critically Important Antimicrobials for Human Medicine.

[4] OIE (2021). OIE List of Antimicrobial Agents of Veterinary Importance.

[5] Partridge S.R., Kwong S.M., Firth N., Jensen S.O. (2018). Mobile genetic elements associated with antimicrobial resistance. Clin. Microbiol. Rev..

[6] Stokes H.W., Gillings M.R. (2011). Gene flow, mobile genetic elements and the recruitment of antibiotic resistance genes into Gram-negative pathogens. FEMS Microbiol. Rev..

[7] Bahadin J., Teo S., Mathew S. (2011). Aetiology of community-acquired urinary tract infection and antimicrobial susceptibility patterns of uropathogens isolated. Singap. Med. J..

[8] Sligl W.I., Dragan T., Smith S.W. (2015). Nosocomial Gram-negative bacteremia in intensive care: Epidemiology, antimicrobial susceptibilities, and outcomes. Int. J. Infect. Dis..

[9] Kaper J.B., Nataro J.P., Mobley H.L. (2004). Pathogenic *Escherichia coli*. Nat. Rev. Microbiol..

[10] Köhler C.D., Dobrindt U. (2011). What defines extraintestinal pathogenic *Escherichia coli*?. Int. J. Med. Microbiol..

[11] Walker C.L.F., Sack D., Black R.E. (2010). Etiology of diarrhea in older children, adolescents and adults: A systematic review. PLoS Negl. Trop. Dis..

[12] Dubreuil J.D., Isaacson R.E., Schifferli D.M. (2016). Animal enterotoxigenic *Escherichia coli*. EcoSal Plus.

[13] Ho P.L., Lo W.U., Lai E.L., Law P.Y., Leung S.M., Wang Y., Chow K.H. (2015). Clonal diversity of CTX-M-producing, multidrug-resistant *Escherichia coli* from rodents. J. Med. Microbiol..

[14] Hasan B., Olsen B., Alam A., Akter L., Melhus Å. (2015). Dissemination of the multidrug-resistant extended-spectrum β-lactamase-producing *Escherichia coli* O25b-ST131 clone and the role of house crow (*Corvus splendens*) foraging on hospital waste in Bangladesh. Clin. Microbiol. Infect..

[15] Mora A., García-Peña F.J., Alonso M.P., Pedraza-Diaz S., Ortega-Mora L.M., Garcia-Parraga D., López C., Viso S., Dahbi G., Marzoa J. (2018). Impact of human-associated *Escherichia coli* clonal groups in Antarctic pinnipeds: Presence of ST73, ST95, ST141 and ST131. Sci. Rep..

[16] Nesporova K., Wyrsch E.R., Valcek A., Bitar I., Chaw K., Harris P., Hrabak J., Literak I., Djordjevic S.P., Dolejska M. (2020). *Escherichia coli* Sequence Type 457 Is an Emerging Extended-Spectrum-β-Lactam-Resistant Lineage with Reservoirs in Wildlife and Food-Producing Animals. Antimicrob. Agents Chemother..

[17] Fulham M., McDougall F., Power M., McIntosh R.R., Gray R. (2022). Carriage of antibiotic resistant bacteria in endangered and declining Australian pinniped pups. PLoS ONE.

[18] Delport T.C., Harcourt R.G., Beaumont L.J., Webster K.N., Power M.L. (2015). Molecular detection of antibiotic-resistance determinants in *Escherichia coli* isolated from the endangered Australian sea lion (*Neophoca cinerea*). J. Wildl. Dis..

[19] McDougall F.K., Boardman W.S., Power M.L. (2021). Characterization of beta-lactam-resistant *Escherichia coli* from Australian fruit bats indicates anthropogenic origins. Microb. Genom..

[20] Sherley M., Gordon D.M., Collignon P.J. (2000). Variations in antibiotic resistance profile in Enterobacteriaceae isolated from wild Australian mammals. Environ. Microbiol..

[21] Lundbäck I.C., McDougall F.K., Dann P., Slip D.J., Gray R., Power M.L. (2021). Into the sea: Antimicrobial resistance determinants in the microbiota of little penguins (*Eudyptula minor*). Infect. Genet. Evol..

[22] Blyton M.D., Pi H., Vangchhia B., Abraham S., Trott D.J., Johnson J.R., Gordon D.M. (2015). Genetic structure and antimicrobial resistance of *Escherichia coli* and cryptic clades in birds with diverse human associations. Appl. Environ. Microbiol..

[23] Mukerji S., Stegger M., Truswell A.V., Laird T., Jordan D., Abraham R.J., Harb A., Barton M., O’Dea M., Abraham S. (2019). Resistance to critically important antimicrobials in Australian silver gulls (*Chroicocephalus novaehollandiae*) and evidence of anthropogenic origins. J. Antimicrob. Chemother..

[24] Mukerji S., Gunasekera S., Dunlop J.N., Stegger M., Jordan D., Laird T., Abraham R.J., Barton M., O’Dea M., Abraham S. (2020). Implications of foraging and interspecies interactions on carriage of *Escherichia coli* resistant to critically important antimicrobials in birds. Appl. Environ. Microbiol..

[25] Smith H.G., Bean D.C., Clarke R.H., Loyn R., Larkins J.A., Hassell C., Greenhill A.R. (2022). Presence and antimicrobial resistance profiles of *Escherichia coli, Enterococcus* spp. and *Salmonella* sp. in 12 species of Australian shorebirds and terns. Zoonoses Public Health.

[26] Wyrsch E.R., Nesporova K., Tarabai H., Jamborova I., Bitar I., Literak I., Dolejska M., Djordjevic S.P. (2022). Urban Wildlife Crisis: Australian Silver Gull Is a Bystander Host to Widespread Clinical Antibiotic Resistance. Msystems.

[27] Skurnik D., Ruimy R., Andremont A., Amorin C., Rouquet P., Picard B., Denamur E. (2006). Effect of human vicinity on antimicrobial resistance and integrons in animal faecal *Escherichia coli*. J. Antimicrob. Chemother..

[28] Mo S.S., Urdahl A.M., Madslien K., Sunde M., Nesse L.L., Slettemeås J.S., Norström M. (2018). What does the fox say? Monitoring antimicrobial resistance in the environment using wild red foxes as an indicator. PLoS ONE.

[29] Arnold K.E., Williams N.J., Bennett M. (2016). ‘Disperse abroad in the land’: The role of wildlife in the dissemination of antimicrobial resistance. Biol. Lett..

[30] Kinjo T., Minamoto N., Sugiyama M., Sugiyama Y. (1992). Comparison of antimicrobial resistant *Escherichia coli* in wild and captive Japanese serows. J. Vet. Med. Sci..

[31] Burgin C.J., Colella J.P., Kahn P.L., Upham N.S. (2018). How many species of mammals are there?. J. Mammal..

[32] Nguema M., Philippe P., Onanga R., Atome N., Roger G., Mbeang O., Constant J., Mabika Mabika A., Yaro M., Lounnas M. (2020). Characterization of ESBL-Producing Enterobacteria from Fruit Bats in an Unprotected Area of Makokou, Gabon. Microorganisms.

[33] Oluduro A.O. (2012). Antibiotic-resistant commensal *Escherichia coli* in faecal droplets from bats and poultry in Nigeria. Vet. Ital..

[34] Benavides J.A., Godreuil S., Opazo-Capurro A., Mahamat O.O., Falcon N., Oravcova K., Streicker D.G., Shiva C. (2022). Long-term maintenance of multidrug-resistant *Escherichia coli* carried by vampire bats and shared with livestock in Peru. Sci. Total Environ..

[35] Garcês A., Correia S., Amorim F., Pereira J.E., Igrejas G., Poeta P. (2019). First report on extended-spectrum beta-lactamase (ESBL) producing *Escherichia coli* from European free-tailed bats (*Tadarida teniotis*) in Portugal: A one-health approach of a hidden contamination problem. J. Hazard. Mater..

[36] Obodoechi L.O., Carvalho I., Chenouf N.S., Martínez-Álvarez S., Sadi M., Nwanta J.A., Chah K.F., Torres C. (2021). Antimicrobial resistance in *Escherichia coli* isolates from frugivorous (*Eidolon helvum*) and insectivorous (*Nycteris hispida*) bats in Southeast Nigeria, with detection of CTX-M-15 producing isolates. Comp. Immunol. Microbiol. Infect. Dis..

[37] Australian_Government (2021). National Recovery Plan for the Grey-Headed Flying Fox Pteropus Poliocephalus. https://www.awe.gov.au/environment/biodiversity/threatened/publications/recovery/grey-headed-flying-fox.

[38] Parry-Jones K., Augee M. (2001). Factors affecting the occupation of a colony site in Sydney, New South Wales by the grey-headed flying-fox *Pteropus poliocephalus* (Pteropodidae). Austral Ecol..

[39] Parris K.M., Hazell D.L. (2005). Biotic effects of climate change in urban environments: The case of the grey-headed flying-fox (*Pteropus poliocephalus*) in Melbourne, Australia. Biol. Conserv..

[40] Boardman W.S., Roshier D., Reardon T., Burbidge K., McKeown A., Westcott D.A., Caraguel C.G., Prowse T.A. (2021). Spring foraging movements of an urban population of grey-headed flying foxes (*Pteropus poliocephalus*). J. Urban Ecol..

[41] Australian_Government (2021). National Flying-Fox Monitoring Programme. http://www.environment.gov.au/biodiversity/threatened/species/flying-fox-monitoring.

[42] Nelson J.E. (1965). Behaviour of Australian pteropodidae (Megacheroptera). Anim. Behav..

[43] Pinson D. (2020). The Flying Fox Manual: A Handbook for Bat Carers.

[44] Clermont O., Christenson J.K., Denamur E., Gordon D.M. (2013). The Clermont *Escherichia coli* phylo-typing method revisited: Improvement of specificity and detection of new phylo-groups. Environ. Microbiol. Rep..

[45] McDougall F., Boardman W., Gillings M., Power M. (2019). Bats as reservoirs of antibiotic resistance determinants: A survey of class 1 integrons in Grey-headed Flying Foxes (*Pteropus poliocephalus*). Infect. Genet. Evol..

[46] Dawes F.E., Kuzevski A., Bettelheim K.A., Hornitzky M.A., Djordjevic S.P., Walker M.J. (2010). Distribution of class 1 integrons with IS*26-*mediated deletions in their 3′-conserved segments in *Escherichia coli* of human and animal origin. PLoS ONE.

[47] Versalovic J., Koeuth T., Lupski R. (1991). Distribution of repetitive DNA sequences in eubacteria and application to fingerprinting of bacterial genomes. Nucleic Acids Res..

[48] Adamus-Bialek W., Wojtasik A., Majchrzak M., Sosnowski M., Parniewski P. (2009). (CGG) 4-based PCR as a novel tool for discrimination of uropathogenic *Escherichia coli* strains: Comparison with enterobacterial repetitive intergenic consensus-PCR. J. Clin. Microbiol..

[49] Bankevich A., Nurk S., Antipov D., Gurevich A.A., Dvorkin M., Kulikov A.S., Lesin V.M., Nikolenko S.I., Pham S., Prjibelski A.D. (2012). SPAdes: A new genome assembly algorithm and its applications to single-cell sequencing. J. Comput. Biol..

[50] Roer L., Tchesnokova V., Allesøe R., Muradova M., Chattopadhyay S., Ahrenfeldt J., Thomsen M.C., Lund O., Hansen F., Hammerum A.M. (2017). Development of a web tool for *Escherichia coli* subtyping based on *fimH* alleles. J. Clin. Microbiol..

[51] Beghain J., Bridier-Nahmias A., Le Nagard H., Denamur E., Clermont O. (2018). ClermonTyping: An easy-to-use and accurate in silico method for *Escherichia* genus strain phylotyping. Microb. Genom..

[52] Zhou Z., Alikhan N.F., Mohamed K., Fan Y., Achtman M., Brown D., Chattaway M., Dallman T., Delahay R., Kornschober C. (2020). The EnteroBase user’s guide, with case studies on *Salmonella* transmissions, *Yersinia pestis* phylogeny, and *Escherichia* core genomic diversity. Genome Res..

[53] Reid C.J., Cummins M.L., Börjesson S., Brouwer M.S., Hasman H., Hammerum A.M., Roer L., Hess S., Berendonk T., Nešporová K. (2022). A role for ColV plasmids in the evolution of pathogenic *Escherichia coli* ST58. Nat. Commun..

[54] Bortolaia V., Kaas R.S., Ruppe E., Roberts M.C., Schwarz S., Cattoir V., Philippon A., Allesoe R.L., Rebelo A.R., Florensa A.F. (2020). ResFinder 4.0 for predictions of phenotypes from genotypes. J. Antimicrob. Chemother..

[55] Carattoli A., Zankari E., García-Fernández A., Larsen M.V., Lund O., Villa L., Aarestrup F.M., Hasman H. (2014). In silico detection and typing of plasmids using PlasmidFinder and plasmid multilocus sequence typing. Antimicrob. Agents Chemother..

[56] Joensen K.G., Scheutz F., Lund O., Hasman H., Kaas R.S., Nielsen E.M., Aarestrup F.M. (2014). Real-time whole-genome sequencing for routine typing, surveillance, and outbreak detection of verotoxigenic *Escherichia coli*. J. Clin. Microbiol..

[57] Chen L., Zheng D., Liu B., Yang J., Jin Q. (2016). VFDB 2016: Hierarchical and refined dataset for big data analysis—10 years on. Nucleic Acids Res..

[58] Johnson J.R., Kuskowski M.A., Owens K., Gajewski A., Winokur P.L. (2003). Phylogenetic origin and virulence genotype in relation to resistance to fluoroquinolones and/or extended-spectrum cephalosporins and cephamycins among *Escherichia coli* isolates from animals and humans. J. Infect. Dis..

[59] Pitout J. (2012). Extraintestinal pathogenic *Escherichia coli*: A combination of virulence with antibiotic resistance. Front. Microbiol..

[60] Matuschek E., Brown D.F., Kahlmeter G. (2014). Development of the EUCAST disk diffusion antimicrobial susceptibility testing method and its implementation in routine microbiology laboratories. Clin. Microbiol. Infect..

[61] Magiorakos A.P., Srinivasan A., Carey R., Carmeli Y., Falagas M., Giske C., Harbarth S., Hindler J., Kahlmeter G., Olsson-Liljequist B. (2012). Multidrug-resistant, extensively drug-resistant and pandrug-resistant bacteria: An international expert proposal for interim standard definitions for acquired resistance. Clin. Microbiol. Infect..

[62] Zhou Z., Alikhan N.F., Sergeant M.J., Luhmann N., Vaz C., Francisco A.P., Carriço J.A., Achtman M. (2018). GrapeTree: Visualization of core genomic relationships among 100,000 bacterial pathogens. Genome Res..

[63] Liu J., Taft D.H., Maldonado-Gomez M.X., Johnson D., Treiber M.L., Lemay D.G., DePeters E.J., Mills D.A. (2019). The fecal resistome of dairy cattle is associated with diet during nursing. Nat. Commun..

[64] Adlerberth I., Wold A. (2009). Establishment of the gut microbiota in Western infants. Acta Paediatr..

[65] Pärnänen K., Karkman A., Hultman J., Lyra C., Bengtsson-Palme J., Larsson D., Rautava S., Isolauri E., Salminen S., Kumar H. (2018). Maternal gut and breast milk microbiota affect infant gut antibiotic resistome and mobile genetic elements. Nat. Commun..

[66] Fulham M., Power M., Gray R. (2018). Comparative ecology of *Escherichia coli* in endangered Australian sea lion (*Neophoca cinerea*) pups. Infect. Genet. Evol..

[67] Lebeaux R.M., Coker M.O., Dade E.F., Palys T.J., Morrison H.G., Ross B.D., Baker E.R., Karagas M.R., Madan J.C., Hoen A.G. (2021). The infant gut resistome is associated with *E. coli* and early-life exposures. BMC Microbiol..

[68] Berge A.C., Hancock D.D., Sischo W.M., Besser T.E. (2010). Geographic, farm, and animal factors associated with multiple antimicrobial resistance in fecal *Escherichia coli* isolates from cattle in the western United States. J. Am. Vet. Med. Assoc..

[69] Hoyle D.V., Shaw D.J., Knight H.I., Davison H.C., Pearce M.C., Low J.C., Gunn G.J., Woolhouse M.E. (2004). Age-related decline in carriage of ampicillin-resistant *Escherichia coli* in young calves. Appl. Environ. Microbiol..

[70] Yun J., Muurinen J., Nykäsenoja S., Seppä-Lassila L., Sali V., Suomi J., Tuominen P., Joutsen S., Hämäläinen M., Olkkola S. (2021). Antimicrobial use, biosecurity, herd characteristics, and antimicrobial resistance in indicator *Escherichia coli* in ten Finnish pig farms. Prev. Vet. Med..

[71] Massot M., Châtre P., Condamine B., Métayer V., Clermont O., Madec J.Y., Denamur E., Haenni M. (2021). Interplay between Bacterial Clones and Plasmids in the Spread of Antibiotic Resistance Genes in the Gut: Lessons from a Temporal Study in Veal Calves. Appl. Environ. Microbiol..

[72] Eby P. (1991). Seasonal movements of grey-headed flying-foxes, *Pteropus poliocephalus* (Chiroptera: Pteropodidae), from two maternity camps in northern New South Wales. Wildl. Res..

[73] Mühldorfer K. (2013). Bats and bacterial pathogens: A review. Zoonoses Public Health.

[74] BOM (2022). Climate Data Online.

[75] Mosley L.M. (2015). Drought impacts on the water quality of freshwater systems; review and integration. Earth-Sci. Rev..

[76] Snoyman S., Muhic J., Brown C. (2012). Nursing females are more prone to heat stress: Demography matters when managing flying-foxes for climate change. Appl. Anim. Behav. Sci..

[77] Suprihatin I., Fallowfield H., Bentham R., Cromar N. (2003). Determination of faecal pollutants in Torrens and Patawalonga catchment waters in South Australia using faecal sterols. Water Sci. Technol..

[78] Brookes J. (2013). River Torrens Water Quality Improvement Trial-Summer 2012/13.

[79] Government_of_South_Australia (2022). Data SA—South Australian Government Data Directory. https://data.sa.gov.au/data/dataset/.

[80] Ahmed A.M., Motoi Y., Sato M., Maruyama A., Watanabe H., Fukumoto Y., Shimamoto T. (2007). Zoo animals as reservoirs of gram-negative bacteria harboring integrons and antimicrobial resistance genes. Appl. Environ. Microbiol..

[81] Medvecky M., Papagiannitsis C.C., Wyrsch E.R., Bitar I., Cummins M.L., Djordjevic S.P., Dolejska M. (2022). Interspecies transmission of CMY-2-producing *Escherichia coli* sequence type 963 isolates between humans and gulls in Australia. Msphere.

[82] Harris P.N., Ben Zakour N.L., Roberts L.W., Wailan A.M., Zowawi H.M., Tambyah P.A., Lye D.C., Jureen R., Lee T.H., Yin M. (2018). Whole genome analysis of cephalosporin-resistant *Escherichia coli* from bloodstream infections in Australia, New Zealand and Singapore: High prevalence of CMY-2 producers and ST131 carrying *bla*CTX-M-15 and *bla*CTX-M-27. J. Antimicrob. Chemother..

[83] Bodley K., Vogelnest L., Portas T. (2019). Appendix 4. Drug formulary. Current Therapy in Medicine of Australian Mammals.

[84] Power M., Vogelnest L., Portas T. (2019). Chapter 17. Antimicrobial resistance. Current Therapy in Medicine of Australian Mammals.

[85] Stecher B., Maier L., Hardt W.D. (2013). ‘Blooming’ in the gut: How dysbiosis might contribute to pathogen evolution. Nat. Rev. Microbiol..

[86] Mo M., Roache M., Davies J., Hopper J., Pitty H., Foster N., Guy S., Parry-Jones K., Francis G., Koosmen A. (2021). Estimating flying-fox mortality associated with abandonments of pups and extreme heat events during the austral summer of 2019–20. Pac. Conserv. Biol..

[87] Mo M., Roache M., Haering R., Kwok A. (2020). Using wildlife carer records to identify patterns in flying-fox rescues: A case study in New South Wales, Australia. Pac. Conserv. Biol.

