# Supplementary material for: High Prevalence of Beta-Lactam-Resistant Escherichia coli in South Australian Grey-Headed Flying Fox Pups (Pteropus poliocephalus)
Source: Microorganisms. 2022 Aug 7;10(8):1589. doi: 10.3390/microorganisms10081589 (PMC9416314; doi:10.3390/microorganisms10081589)
Supplement: Supplementary file 1 [file microorganisms-10-01589-s001.zip › MICROORGANISMS 1818722 _Supplementary Tables S1 to S5.pdf]

Supplementary Table S1. Strain typing of 49 amoxicillin resistant *Escherichia coli* isolates from grey-headed flying fox pups using a combination of phylotyping, genome fingerprints (ERIC and CGG) and class 1 integron PCR data.

Isolate ID	PG PCR	ERIC PCR type	CGG PCR type	Integron Integrase PCR	Integron gene cassette PCRs	WGS
FF1568B1	A	A-1	A-1	Negative	-	Yes
FF1563	A/C	A/C-1	A/C-1	Negative	-	Yes
FF1575	A/C	A/C-1	A/C-1	Negative	-	No
FF1576A2	A/C	A/C-1	A/C-1	Negative	-	No
FF1565	A/C	A/C-2	A/C-2	Negative	-	Yes
FF1566	A/C	A/C-2	A/C-2	Negative	-	No
FF1600	A/C	A/C-2	A/C-2	Negative	-	No
FF1568A	A/C	A/C-3	A/C-3	Negative	-	Yes
FF1584B	A/C	A/C-3	A/C-3	Negative	-	No
FF1570A	A/C	A/C-4	A/C-4	Positive	HS458/JL-D2 positive (1 band, ~2kbp)	Yes
FF1571	A/C	A/C-4	A/C-4	Positive	HS458/JL-D2 positive (1 band, ~2kbp)	No
FF1576A1-2	A/C	A/C-4	A/C-4	Positive	HS458/JL-D2 positive (1 band, ~2kbp)	No
FF1585B	A/C	A/C-4	A/C-4	Positive	HS458/JL-D2 positive (1 band, ~2kbp)	No
FF1589	A/C	A/C-4	A/C-4	Positive	HS458/JL-D2 positive (1 band, ~2kbp)	No
FF1570B1	A/C	A/C-5	A/C-5	Negative	-	Yes
FF1397	B1	B1-1	B1-1	Negative	-	No
FF1410	B1	B1-1	B1-1	Negative	-	No
FF1412	B1	B1-1	B1-1	Negative	-	No
FF1418	B1	B1-1	B1-1	Negative	-	No
FF1419	B1	B1-1	B1-1	Negative	-	No
FF1532	B1	B1-1	B1-1	Negative	-	Yes
FF1533	B1	B1-1	B1-1	Negative	-	No
FF1534	B1	B1-1	B1-1	Negative	-	No
FF1535	B1	B1-1	B1-1	Negative	-	No
FF1536	B1	B1-1	B1-1	Negative	-	No
FF1562	B1	B1-2	B1-2	Negative	-	Yes
FF1572	B1	B1-3	B1-3	Negative	-	Yes
FF1576A1-1	B1	B1-3	B1-3	Negative	-	No
FF1598	B1	B1-4	B1-4	Negative	-	Yes
FF1582	B1	B1-5	B1-5	Positive	HS458/JL-D2 positive (3 bands, >800bp)	Yes
FF1583	B1	B1-5	B1-5	Positive	HS458/JL-D2 positive (3 bands, >800bp)	No
FF1584A	B1	B1-5	B1-5	Positive	HS458/JL-D2 positive (3 bands, >800bp)	No
FF1586	B1	B1-5	B1-5	Positive	HS458/JL-D2 positive (3 bands, >800bp)	No
FF1587	B1	B1-5	B1-5	Positive	HS458/JL-D2 positive (3 bands, >800bp)	No
FF1588	B1	B1-5	B1-5	Positive	HS458/JL-D2 positive (3 bands, >800bp)	No
FF1590	B1	B1-5	B1-5	Positive	HS458/JL-D2 positive (3 bands, >800bp)	No
FF1591	B1	B1-5	B1-5	Positive	HS458/JL-D2 positive (3 bands, >800bp)	No
FF1593	B1	B1-5	B1-5	Positive	HS458/JL-D2 positive (3 bands, >800bp)	No
FF1594	B1	B1-5	B1-5	Positive	HS458/JL-D2 positive (3 bands, >800bp)	No
FF1595	B1	B1-5	B1-5	Positive	HS458/JL-D2 positive (3 bands, >800bp)	No
FF1596	B1	B1-5	B1-5	Positive	HS458/JL-D2 positive (3 bands, >800bp)	No
FF1597	B1	B1-5	B1-5	Positive	HS458/JL-D2 positive (3 bands, >800bp)	No
FF1599	B1	B1-5	B1-5	Positive	HS458/JL-D2 positive (3 bands, >800bp)	No
FF1567	D/E	D/E-1	D/E-1	Negative	-	Yes
FF1569	D/E	D/E-1	D/E-1	Negative	-	No
FF1573	D/E	D/E-1	D/E-1	Negative	-	No
FF1574	D/E	D/E-1	D/E-1	Negative	-	No
FF1579	D/E	D/E-1	D/E-1	Negative	-	No
FF1581	D/E	D/E-1	D/E-1	Negative	-	No

Supplementary Table S2. Individual BioSample accession numbers under NCBI Sequence Read Archive (SRA) BioProject ID PRJNA814458, EnteroBase Barcodes and GenBank accession numbers for class 1 integrons from 12 antimicrobial resistant *Escherichia coli* isolated from grey-headed flying fox pups in care.

Isolate ID	ST	Serotype	BioSample No.	EnteroBase Barcode	Integron GenBank accession No.
FF1563	10	ONT:H32	SAMN26553891	ESC_UA2847AA	-
FF1570A	48	O4:H26	SAMN26553892	ESC_UA2853AA	ON087280
FF1570B1	48	B18:H11	SAMN26553893	ESC_UA2854AA	-
FF1582	58	O8:H25	SAMN26553894	ESC_UA2849AA	ON087281
FF1598	58	ONT:H37	SAMN26553895	ESC_UA2856AA	-
FF1568A	361	O9:H30	SAMN26553896	ESC_UA2851AA	-
FF1562	641	O70:H10	SAMN26553897	ESC_UA2846AA	-
FF1565	710	B9:H30	SAMN26553898	ESC_UA2848AA	-
FF1567	963	ONT:H18	SAMN26553899	ESC_UA2850AA	-
FF1568B1	1421	O9:H4	SAMN26553900	ESC_UA2852AA	-
FF1572	1727	ONT:H14	SAMN26553901	ESC_UA2855AA	-
FF1532	2144	O166:H49	SAMN26553902	ESC_UA2845AA	-

Supplementary Table S3. Antibiotic discs used for EUCAST susceptibility testing.

Antibiotic and disc content (µg)	OXOID disc	Antibiotic category and generation	Breakpoint
Ampicillin (10 µg)	AMP10	ß-lactam/Penicillins	EUCAST
Amoxicillin/Clavulanic Acid (30 µg)	AMC30	ß-lactam/Penicillins + ß-lactamase inhibitors	EUCAST
Cephalexin (30 µg)	CL30	ß-lactam/Cephalosporins/1st Generation (Non-extended spectrum cephalosporin)	EUCAST
Cefotaxime (5 µg)	CTX5	ß-lactam/Cephalosporins/3rd Generation (Extended-spectrum cephalosporin)	EUCAST
Imipenem (10 µg)	IPM10	ß-lactam/Carbapenems	EUCAST
Nalidixic acid (30 µg)	NA30	Quinolones-Fluoroquinolones/ 1st Generation Quinolone	CLSI
Trimethoprim (5 µg)	W5	Trimethoprim (Folate pathway inhibitors)	EUCAST
Trimethoprim/Sulfamethoxazole (30 µg)	SXT30	Trimethoprim + Sulfonamide (Folate pathway inhibitors)	EUCAST
Amikacin (30 µg)	AK30	Aminoglycosides	EUCAST
Gentamycin (10 µg)	CN10	Aminoglycosides	EUCAST
Streptomycin (25 µg)	S25	Aminoglycosides	NA*
Spectinomycin (25 µg)	SH25	Aminoglycosides	NA*
Chloramphenicol (30 µg)	C30	Phenicols	EUCAST
Nitrofurantoin (200 µg)	F200	Nitrofurantoin	NA*
Tetracycline (30 µg)	TE30	Tetracyclines	CLSI

* NA = Not Available: Breakpoint criteria unavailable from EUCAST or CLSI tables.

Supplementary Table S4. URL links to interactive versions of GrapeTree cgMLST phylogeny trees for GHFF pup and closely related *E. coli* isolates in EnteroBase.

ST and serotype	URL link
ST 10 ONT:H32	https://achtman-lab.github.io/GrapeTree/MSTree_holder.html?tree=https://github.com/Fiona-McDougall/GHFF-Phylogenetics/blob/master/ST10_ONT_H32_n=39_ms_tree.json
ST48 O4:H26	https://achtman-lab.github.io/GrapeTree/MSTree_holder.html?tree=https://github.com/Fiona-McDougall/GHFF-Phylogenetics/blob/master/ST48_O4_H26_n=122_ms_tree.json
ST48 B18:H11	https://achtman-lab.github.io/GrapeTree/MSTree_holder.html?tree=https://github.com/Fiona-McDougall/GHFF-Phylogenetics/blob/master/ST48_B18_H11_n=166_ms_tree.json
ST58 O8:H25	https://achtman-lab.github.io/GrapeTree/MSTree_holder.html?tree=https://github.com/Fiona-McDougall/GHFF-Phylogenetics/blob/master/ST58_O8_H25_n=439_ms_tree.json
ST58 ONT:H37	https://achtman-lab.github.io/GrapeTree/MSTree_holder.html?tree=https://github.com/Fiona-McDougall/GHFF-Phylogenetics/blob/master/ST58_ONT_H37_n=130_ms_tree.json
ST361 O9:H30	https://achtman-lab.github.io/GrapeTree/MSTree_holder.html?tree=https://github.com/Fiona-McDougall/GHFF-Phylogenetics/blob/master/ST361_O9_H30_n=323_ms_tree.json
ST641 O70:H10	https://achtman-lab.github.io/GrapeTree/MSTree_holder.html?tree=https://github.com/Fiona-McDougall/GHFF-Phylogenetics/blob/master/ST641_O70_H10_n=40_ms_tree.json
ST710 B9:H30	https://achtman-lab.github.io/GrapeTree/MSTree_holder.html?tree=https://github.com/Fiona-McDougall/GHFF-Phylogenetics/blob/master/ST710_B9_H30_n=27_ms_tree.json
ST963 ONT:H18	https://achtman-lab.github.io/GrapeTree/MSTree_holder.html?tree=https://github.com/Fiona-McDougall/GHFF-Phylogenetics/blob/master/ST963_ONT_H18_n=184_ms_tree.json
ST1421 O9:H4	https://achtman-lab.github.io/GrapeTree/MSTree_holder.html?tree=https://github.com/Fiona-McDougall/GHFF-Phylogenetics/blob/master/ST1421_O9_H4_n=76_ms_tree.json
ST1727 ONT:H14	https://achtman-lab.github.io/GrapeTree/MSTree_holder.html?tree=https://github.com/Fiona-McDougall/GHFF-Phylogenetics/blob/master/ST1727_ALL_MINUS_H49_n=153_ms_tree.json
ST2144 O166:H49	https://achtman-lab.github.io/GrapeTree/MSTree_holder.html?tree=https://github.com/Fiona-McDougall/GHFF-Phylogenetics/blob/master/ST2144_O166_H49_n=80_ms_tree.json

Supplementary Table S5. Phenotypic antimicrobial susceptibility testing of 12 *E. coli* strains from grey-headed flying-fox pups. Antibiotic codes: AMP, Ampicillin; AML, Amoxicillin; AMC, Amoxicillin/Clavulanic acid; CL, Cephalexin; CTX, Cefotaxime; IPM, Imipenem; NA, Nalidixic acid; W, Trimethoprim; SXT, Trimethoprim/Sulfamethoxazole; AK, Amikacin; CN, Gentamycin; S, Streptomycin; SH, Spectinomycin; C, Chloramphenicol; F, Nitrofurantoin; TE, Tetracycline. Other abbreviations: ST, Sequence Type; ONT, Onon-typable, R, Resistant; S, Susceptible; I, Intermediate resistance; AMR, Antimicrobial Resistance. Multidrug drug resistance (MDR) is defined as as acquired phenotypic resistance to at least one antimicrobial in three or more AMR categories. Antimicrobial Categories are denoted with superscript numbers: 1. Penicillins; 2. Penicillins + β-lactamase inhibitors; 3. Non-extended spectrum cephalosporins - 1st generation; 4. Extended-spectrum cephalosporins - 3rd generation; 5. Carbapenems; 6. Quinolones; 7. Folate pathway inhibitors; 8. Aminoglycosides; 9. Phenicols; 10. Nitrofurantoin; 11. Tetracyclines. Strains exhibiting MDR are highlighted in bold font.

Strain type	AMP[1]	AML[1]	AMC[2]	CL[3]	CTX[4]	IPM[5]	NA[6]	W[7]	SXT[7]	AK[8]	CN[8]	S[8]	SH[8]	C[9]	F[10]	TE[11]	No. AMR categories
**ST10 ONT:H32**	R	R	S	S	S	S	S	S	S	S	S	I	S	S	S	R	**3**
**ST48 O4:H26**	R	R	S	S	S	S	S	R	R	S	S	S	I	S	S	R	**4**
ST48 B18:H11	R	R	S	S	S	S	S	S	S	S	S	S	S	S	S	S	1
**ST58 O8:H25**	R	R	R	S	S	S	S	R	R	S	S	R	S	S	S	S	**3**
**ST58 ONT:H37**	R	R	S	S	S	S	S	S	S	S	S	S	S	R	S	R	**3**
ST361 O9:H30	R	R	S	S	S	S	S	S	S	S	S	S	S	S	S	R	2
ST641 O70:H10	R	R	S	S	S	S	S	S	S	S	S	S	S	S	S	S	1
ST710 B9:H30	R	R	S	S	S	S	S	S	S	S	S	S	S	S	S	R	2
**ST963 ONT:H18**	R	R	R	R	R	S	S	S	S	S	S	S	S	S	S	S	**3**
ST1421 O9:H4	R	R	S	S	S	S	S	S	S	S	S	S	S	S	S	R	2
ST1727 ONT:H14	R	R	S	S	S	S	S	S	S	S	S	S	S	S	S	R	2
ST2144 O166:H49	R	R	S	S	S	S	S	S	S	S	S	S	S	S	S	S	2
